# MetaboHunter: an automatic approach for identification of metabolites from ^1^H-NMR spectra of complex mixtures

**DOI:** 10.1186/1471-2105-12-400

**Published:** 2011-10-14

**Authors:** Dan Tulpan, Serge Léger, Luc Belliveau, Adrian Culf, Miroslava Čuperlović-Culf

**Affiliations:** 1 Institute for Information Technology, National Research Council of Canada, Moncton, New Brunswick, E1A 7R1, Canada; 2 Atlantic Cancer Research Institute, Moncton, New Brunswick, E1C 8X3, Canada; 3 Department of Chemistry and Biochemistry, Mount Allison University, Sackville, New Brunswick, E4L 1G8, Canada; 4 Department of Biology, Université de Moncton, New Brunswick, E1A 3E9, Canada

## Abstract

**Abstract:**

**Background**

One-dimensional ^1^H-NMR spectroscopy is widely used for high-throughput characterization of metabolites in complex biological mixtures. However, the accurate identification of individual compounds is still a challenging task, particularly in spectral regions with higher peak densities. The need for automatic tools to facilitate and further improve the accuracy of such tasks, while using increasingly larger reference spectral libraries becomes a priority of current metabolomics research.

**Results**

We introduce a web server application, called MetaboHunter, which can be used for automatic assignment of ^1^H-NMR spectra of metabolites. MetaboHunter provides methods for automatic metabolite identification based on spectra or peak lists with three different search methods and with possibility for peak drift in a user defined spectral range. The assignment is performed using as reference libraries manually curated data from two major publicly available databases of NMR metabolite standard measurements (HMDB and MMCD). Tests using a variety of synthetic and experimental spectra of single and multi metabolite mixtures show that MetaboHunter is able to identify, in average, more than 80% of detectable metabolites from spectra of synthetic mixtures and more than 50% from spectra corresponding to experimental mixtures. This work also suggests that better scoring functions improve by more than 30% the performance of MetaboHunter's metabolite identification methods.

**Conclusions**

MetaboHunter is a freely accessible, easy to use and user friendly ^1^H-NMR-based web server application that provides efficient data input and pre-processing, flexible parameter settings, fast and automatic metabolite fingerprinting and results visualization via intuitive plotting and compound peak hit maps. Compared to other published and freely accessible metabolomics tools, MetaboHunter implements three efficient methods to search for metabolites in manually curated data from two reference libraries.

**Availability:**

http://www.nrcbioinformatics.ca/metabohunter/

## Background

High throughput metabolic profiling has been performed for over 40 years [1] on tissue extracts and biofluids. However, due to recent analytical and computational advances, metabolomics, as is now known, is an increasingly popular approach for monitoring multi-parametric responses in complex biological systems with applications ranging from the analysis of unicellular samples all the way to the analysis of complex systems such as plants and mammals. By definition, metabolomics is a comprehensive qualitative and quantitative study of small molecules composition of organisms [2]. NMR spectroscopy is one of the most widely used methods for analytical measurement of metabolic profiles in systems particularly because of its reliability, reproducibility, speed and low cost [3,4].

One of the major challenges in NMR analysis of metabolic profiles is the automatic metabolite assignment from spectra. Current approaches include manual assignment based on user experience and the assignment based on binning, curve-fitting and direct comparison of 1D and 2D NMR measurements [5-7] with and without reference library support. Although the two approaches have their merit, the manual assignment is highly biased towards user knowledge and expectations and 2D methods can be time consuming and yet still insufficient for direct assignment [7]. At the same time, unlike the classical NMR applications in molecular structure identification, in metabolomic applications, molecular structures of common metabolites are already known and thus assignment of spectra can be done by direct comparison with reference libraries, when these become available.

Various approaches were described in previous publications, including: (i) binning approaches [8,9] where a spectrum is typically divided into equally or variable sized bins and the intensities in each bin are qualified and quantified via integration techniques; (ii) curve fitting without reference library support, where de-convoluting highly overlapped linearly mixed individual metabolite spectra is achieved via various methodologies ranging from Bayesian decompositions [10,11] and least squares-based non-negative matrix factorization [12] to shape fitting techniques [13-17]; (iii) curve fitting with reference library support, where least squares strategies [18-20], Bayesian model selection [21], and genetic algorithms [22,23] are employed, and (iv) direct comparison methods that calculate the overlap of known peaks with peaks from query spectra [22]. More comprehensive descriptions of methods and practical aspects of applied metabolomics are described in a number of recent publications [24-27].

Two large collections of ^1^H-NMR spectra of known metabolites are already available as part of the Human Metabolome Database - HMDB [28] and Madison Metabolomics Consortium Database - MMCD [29]. Thus, for experiments performed under similar conditions as the ones used for creating the reference library, a necessary step for automatic assignment is the development of an efficient bioinformatic tool for comparison, identification and assignment directly from the spectra.

In this paper we introduce MetaboHunter - an efficient automatic metabolite identification approach that provides three distinct methods and two reference libraries wrapped up in an intuitive, flexible and user friendly web server application. The three methods include: (i) a novel scoring function that outperforms the simpler peaks match percentages employed by other tools such as HMDB NMR Search [28] and MetaboMiner [6], (ii) an iterative greedy selective approach that minimizes the number of false positives at the expense of slightly increasing the number of false negatives and, (iii) selection approaches with a user adjustable chemical peak drift parameter that allows the identification of mixture metabolites in spectra obtained under slightly different conditions than the ones used for the measurement of reference library compounds. In addition to what other metabolite selection strategies have to offer, our approach includes a simple and intuitive graphical user interface, two manually curated reference libraries of metabolite spectra, a better metabolite scoring function and a set of three selection strategies that, in average, outperform existing methods provided by other publicly available metabolite identification software.

## Results and Discussion

### System Architecture

MetaboHunter is a web-server application for identification of metabolites in ^1^H-NMR spectra of complex mixtures based on two manually curated reference libraries. The individual spectra of metabolites from the reference libraries and the additional metabolite information are stored in plain text files and are also indexed based on their peak locations and original IDs for faster access. The user interaction is performed via an intuitive and user-friendly graphical interface powered by PHP and JavaScript routines. MetaboHunter takes as input a full spectrum or a list of peaks and their corresponding measured amplitudes, which can be either provided as text files or pasted directly into a text box. Once the input is provided, a session is created for each user and the index files are uploaded. The full spectrum input files are pre-screened based on the noise threshold using custom-built Perl scripts and one of the three metabolite selection methods are executed. All three methods are also implemented in Perl. The output consisting of a list of potential metabolite matches is presented to the user and further functionality is provided to visualize, compare and download the results. More details regarding the pre-screening process, the metabolite selection methods and the reference libraries are presented in the Methods Section.

### MetaboHunter Testing

Different NMR spectra, combinations of molecular spectra as well as spectra of a chemical mixture were used for testing the methods provided as part of MetaboHunter. Validation was performed in all cases by analyzing results of searches using complete, not binned spectra as well as externally determined peak lists. Peaks from FID files were determined using MNova software by Mestrelab Research http://www.mestrec.com. The Human Metabolomics Database (HMDB) contains different information about many metabolites including their NMR chemical shifts and these were used for the majority of tests presented here. In the test examples shown below we have included metabolites with large and small number of spectral peaks where some peaks were non-unique. Results of the search for spectra of several individual metabolites (SYN1 - SYN4) are shown in Additional file 1, Table 1S. For the analysis of MetaboHunter's ability to assign metabolites from the spectra of mixtures we have combined free induction decay (FID) measurements of 13 metabolites provided by HMDB into one NMR spectrum. Assuming that there are no chemical interactions between molecules, the NMR spectrum of a mixture is a direct sum of spectra of the components. For this test we have selected 13 water soluble, *i.e. *hydrophilic metabolites that were consistently observed in standard 1D ^1^H-NMR of human cell lines [30-34]. The results of assignments for this spectrum are presented in Additional file 1, Table 2S. Finally, MetaboHunters's assignment power was investigated against the NMR experimental measurements of a spiked-in urine sample (EXP1) measured on a 500 MHz Bruker Avance NMR spectrometer and a mixture of 5 metabolites (EXP2) performed in house on a 270 MHz JEOL JNM-GSX Nuclear Magnetic Resonance Spectrometer (Mount Allison University). The peak databases used by MetaboHunter include measurements from 400 MHz, 500 MHz and 600 MHz instruments. Thus validation with spectra from the 500 MHz and the 270 MHz shows the possibility of using methods provided under MetaboHunter platform for assignment of data coming from different NMR field strength measurements. The results of this analysis are shown in Additional file 1, Tables 3S and 5S. The results reported in Additional file 1, Table 5S show prediction accuracy for the spiked metabolites and also include a list of 10 additional metabolites, such as Creatinine, Glycine and Lysine, commonly found in urine [35], that were correctly identified with our methods.

### Analysis of metabolite recovery for MH1_HMDB, MH1_MMCD, MH3_HMDB and MH3_MMCD from simulated mixtures of randomly selected library metabolites

A test of the metabolite fingerprinting engine employed in MetaboHunter for the analysis of NMR peak information was performed first by automatic search of peak list mixtures for *n *metabolites, where *n *= 1:10. Each result was averaged over 100 runs, each run consisting of randomly selecting and mixing the peaks for *n *metabolites from HMDB and MMCD. This controlled approach can evaluate the ability of the MH1 and MH3 methods to efficiently recover randomly selected metabolites from the supporting HMDB and MMCD libraries present in various mixtures.

**Figure 1:**
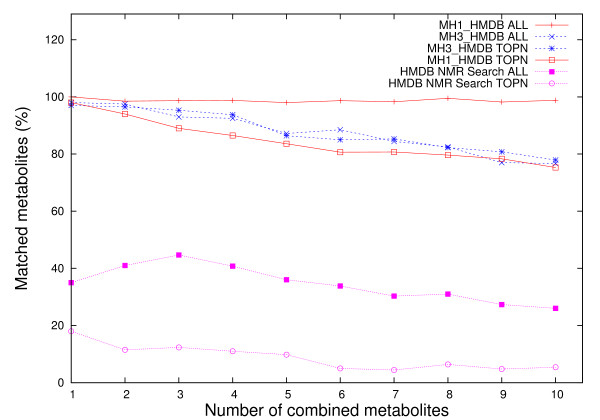
**Results of metabolite searches for mixtures of HMDB peaks corresponding to n metabolites, where n = 1:10**. The curves labelled "MHX_DB ALL", where X = {1,3} and DB = {HMDB, MMCD}, represent average percentages of correctly matched metabolites over 100 runs using MH1 and MH3, when all matches regardless of their position are considered, whereas the curves labelled "MHX_DB TOPN" represent average percentages of correctly matched metabolites over 100 runs using MH1 and MH3, when only top *n *matches were selected, where *n *= 1:10. The "HMDB NMR Search ALL" and "HMDB NMR Search TOPN" curves represent average percentages of correctly matched metabolites over 100 runs using the HMDB NMR Search option available online. These curves are not present in the MMCD plot since metabolites have different names and the database does not have a 100% overlap with HMDB.

**Figure 2:**
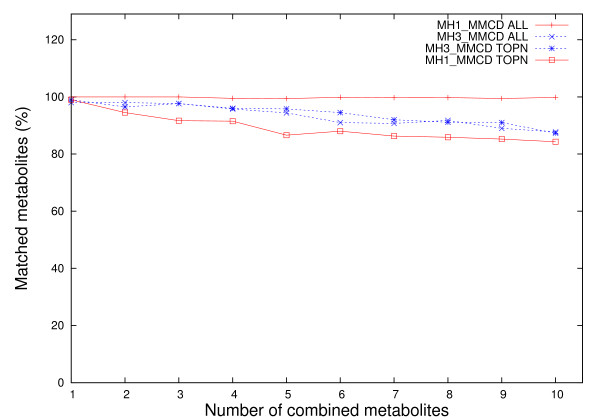
**Results of metabolite searches for mixtures of MMCD peaks corresponding to n metabolites, where n = 1:10**. The curves labelled "MHX_DB ALL", where X = {1,3} and DB = {HMDB, MMCD}, represent average percentages of correctly matched metabolites over 100 runs using MH1 and MH3, when all matches regardless of their position are considered, whereas the curves labelled "MHX_DB TOPN" represent average percentages of correctly matched metabolites over 100 runs using MH1 and MH3, when only top *n *matches were selected, where *n *= 1:10.

The accuracy of this search, *i.e*. its ability to return metabolites present in the mixtures, is automatically determined from investigating whether the metabolites are included: a) in the complete list and b) in the top *n *listed metabolites (Figures 1 and 2). When the complete lists of proposed assignments are analyzed, 100% (MH1_HMDB) and respectively 98% (MH3_HMDB) of metabolites (*n *= 1) in the single component mixture are found, while for mixtures with more than one metabolite (*n > 1*, Figures 1 and 2) the decrease in the average percentage of correctly identified metabolites is insignificant for MH1_HMDB (98.8% for *n *= 10) but becomes more drastic for MH3_HMDB (76.7% for *n *= 10). The same effect is observed for MH1_MMCD and MH3_MMCD. The lower percentage obtained with MH3_HMDB for increasingly large numbers of metabolites in mixtures is caused by the high overlaps in shared peaks among library metabolites and the mechanism for selecting metabolites with disjoint peaks adopted in MH3 (details in Methods). The same analysis reveals that HMDB NMR Search is able to identify only 35% of the metabolites for *n *= 1 and only 26% for *n *= 10.

If only the top *n *scored assignments are included, we observe a minor decrease in accuracy of MH1 and MH3 with the increase of the number of metabolites in the mixture. In this case, over 98% of the time (MH1_HMDB and MH3_HMDB), the top metabolite corresponds to the peak list for *n *= 1 and the accuracy decreases to 75.3% (MH1_HMDB) and 77.9% (MH3_HMDB) for mixtures of *n *= 10 metabolites. For the same type of analysis and the same benchmark data, HMDB NMR Search is able to retrieve 18% of the metabolites for *n *= 1 and only 5.4% when *n *= 10.

### The effects of spectral noise originated from partial peak information and chemical shift variations on metabolite identification

**Figure 3:**
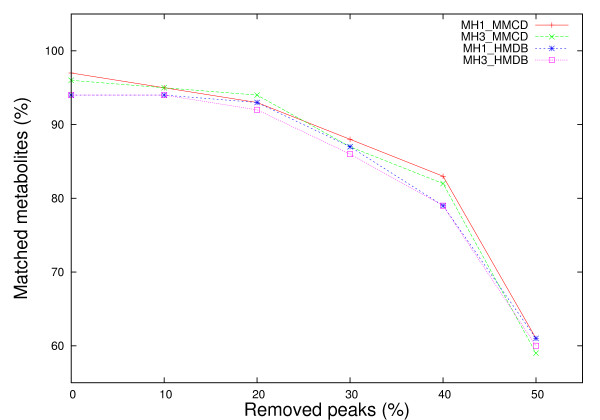
**Comparative performance of metabolite matching strategies applied on individual metabolite spectra with removed peaks**. Each experimental metabolite spectrum from both reference libraries was altered by removing from 0% to 50% of the peaks and then searched against both libraries. The percentage of matched metabolites was averaged over 5 iterations.

We estimated the performance of the proposed methods using data from both spectral libraries under two types of noise - missing peaks (MH1, MH3) and chemically shifted peaks (MH1, MH2, MH3). Following the methodology described by Xia *et al. *[6], we simulated missing peaks by removing from each spectral compound uniformly at random between 0% and 50% of the peaks. The chemical shift variations were simulated by adding random values of ± 0.01, ± 0.02, ± 0.03, ± 0.04 and ± 0.05 ppm to each ^1^H chemical shift. As a first step towards the evaluation of our methods, we applied an exhaustive approach where peaks for each individual metabolite from both reference libraries (HMDB, MMCD) were subject to noise adjustments as described above. We repeated the process 5 times per metabolite and reported the average value. When peaks were removed at random from each metabolite (Figure 3), on average, no major loss of performance is observed (performance is around 95%) for up to 20% of peaks removed. The percentage of correctly identified metabolites decreases with removing more peaks, down to approximately 60% assignment accuracy when half of the peaks are removed from each metabolite spectrum. We also notice that both, MH1 and MH3 methods using HMDB and MMCD reference libraries fare equally well in this case.

**Figure 4:**
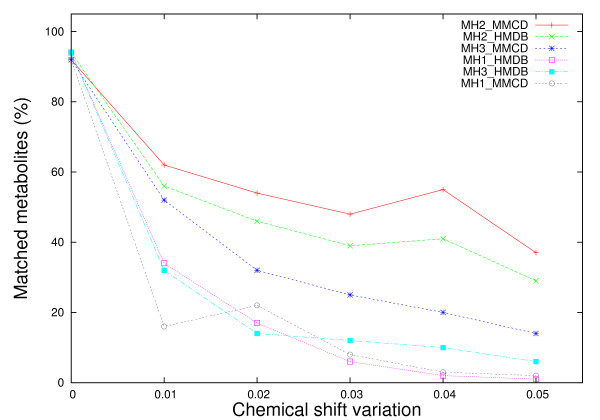
**Comparative performance of metabolite matching strategies applied on individual metabolite spectra with chemical shift variations**. Each experimental metabolite spectrum from both reference libraries were altered by adding/subtracting a chemical shift variation from 0.00 ppm to ± 0.05 ppm in equal increments of 0.01 ppm and then searched against both libraries. The percentage of matched metabolites was averaged over 5 iterations.

Nevertheless, the performance of MH1 and MH3 methods is clearly affected by noise originated from chemical shift variations (Figure 4). For a chemical shift variation of ± 0.01 ppm, MH1 with HMDB and MMCD reference libraries suffers a performance drop from above 90% (no shift) to below 40% (34% for MH1_HMDB, 16% for MH1_MMCD). When the chemical shift variation increases up to ± 0.05 ppm, the performance of MH1 (HMDB and MMCD) drops below 5% (1% and respectively 2%). A similar but not so dramatic drop in performance is also noticed for the MH3 method with HMDB and MMCD reference libraries. For a ± 0.05 ppm chemical shift variation, MH3's performance drops down to 6% (HMDB) and respectively 14% (MMCD).

Figure 4 shows that a large proportion of the performance loss due to chemical shift changes could be recovered if MH2 method is used instead of MH1. Assuming that the chemical shift variation parameter approximates well the true chemical shift change, MH2 recovers the performance lost by MH1 on average up to 30.2% of the time when HMDB is used as reference library and up to 41% when MMCD is the reference library.

**Figure 5:**
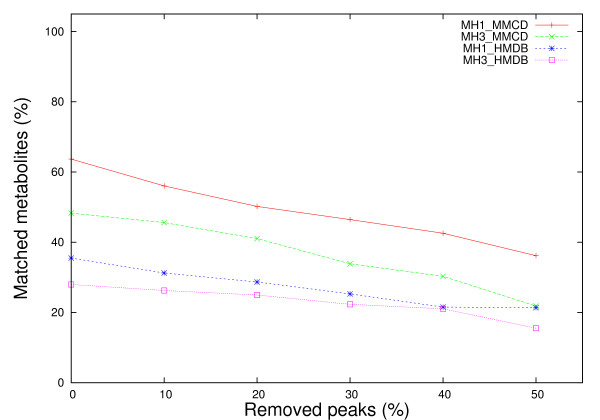
**Comparative performance of metabolite matching strategies applied on synthetic mixtures with removed peaks**. Synthetic mixture spectra were obtained by pooling peaks from 50 randomly selected metabolites from the two reference libraries (HMDB and MMCD). Spectral noise was introduced by removing from 0% to 50% of the peaks. The percentage of correctly identified metabolites was averaged over 50 iterations.

**Figure 6:**
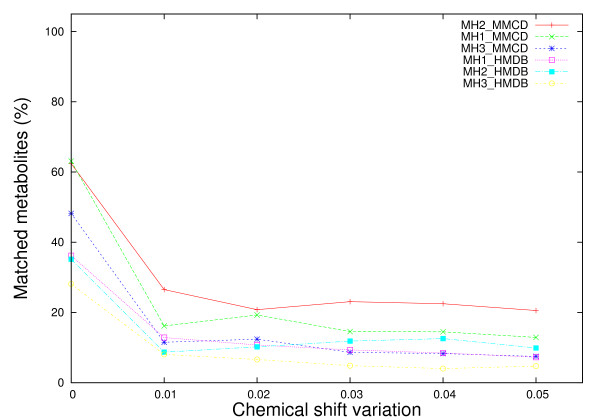
**Comparative performance of metabolite matching strategies applied on synthetic mixtures with chemical shift variations**. Synthetic mixture spectra were obtained by pooling peaks from 50 randomly selected metabolites from the two reference libraries (HMDB and MMCD). Spectral noise was introduced by adding/subtracting a chemical shift variation from 0 ppm to ± 0.05 ppm in equal increments of 0.01. The percentage of correctly identified metabolites was averaged over 50 iterations.

As a second evaluation step, we applied the random pooling technique described in [6], where we generated synthetic mixtures by pooling uniformly at random 50 metabolites from each of the two metabolite reference libraries (HMDB and MMCD). After injecting chemical noise in each metabolite spectrum via chemical shift alterations and random peaks removal, the mixtures were searched against the reference libraries using MH1, MH2 and MH3 and a successful match is recorded if the mixture metabolite was reported within the first 50 results produced by each method. Here, our reporting procedure is more stringent and differs from [6], since they adopted a threshold based approach (75%) for their perfect match method, which in turn does not set a limit on the total number of reported matches. The process was repeated 50 times for each chemical shift variation and peak removal percentage and the average value was reported (Figures 5 and 6). Since the MH2 method was proposed to alleviate the insufficiencies of MH1 caused by chemical shift variations, MH2 was only applied to data that suffered chemical shift modifications.

Figure 5 depicts the same performance decreasing trend of the proposed methods with the increase of the percentage of removed peaks, as it was observed when single metabolites with noisy peak values were searched against reference libraries. In contrast with the results obtained by Xia *et al. *[6], whose percentage match method using complex and more complete 2D spectral information exhibited a sharp decrease in performance (70%), when the percentage of removed peaks varies from 0% to 50%, we notice an average performance decrease of less than 20% for both MH1 and MH3 methods regardless of the reference library being used. As clearly shown in Figure 5, the performance decrease is less stringent for MH1 and MH3 using HMDB spectral data; although the initial performance (35% for 0% peaks removal) is 25% lower compared to the performance when using MMCD spectral data (64% for 0% peaks removal).

Similarly as for the case of single metabolite identification against reference libraries, the noise introduced by chemical shift variations affects the performance of the proposed methods more. Figure 6 shows that 47% performance loss (from 63% to 16%) is recorded for MH1_MMCD, while only 20% performance loss (from 28% to 8%) was recorded for MH3_HMDB. The best performing method overall is MH2_MMCD, which improves the performance of MH1_MMCD with up to 10% (for ± 0.01 chemical shift variation). When compared to the results obtained with MetaboMiner [6], our percentage match - based method, MH1, seems to be affected earlier by smaller chemical shift changes (± 0.01 ppm). Nevertheless its performance does not deteriorate almost at all when larger deviations are applied on the peak locations. This is in stark contrast to the PM method implemented in MetaboMiner, which loses only 20% in performance for deviations in chemical shift of ± 0.02 ppm, but drops by more than 70% down to 10% performance for chemical shift changes of ± 0.04 ppm and ± 0.05 ppm.

Compared to the performance of our methods when single metabolite spectra (noise adjusted) are searched against reference libraries, the relatively large size (50 metabolites) of the mixture combined with the large size of the reference spectral libraries, adds complexity and difficulty to the metabolite identification problem.

### The selection of optimal cut-offs

When a mixture with an unknown number of metabolites is analyzed, the selection of an optimal cut-off point for the metabolite list that will balance specificity and sensitivity and optimize accuracy is important. Here we use Receiver Operating Characteristic (ROC) curves to shed light on cut-offs that seem reasonable for both, synthetic and experimental data.

Each cut-off corresponds to a point on a ROC curve. The ROC curve has the sensitivity plotted vertically and the reversed scale of the specificity on the horizontal axis. The scale of the horizontal axis is also called the false positive rate. The sensitivity and specificity, and therefore the performance of the system, vary with the cut-off. When comparing ROC curves of different methods, good curves lie closer to the top left corner with the diagonal line representing the random solution.

When synthetic data sets containing mixtures of 13 metabolites are considered (SYN5_s, SYN5_f, SYN5_p), the highest accuracies (0.99) are obtained with MH1_HMDB (see Table 1 and Additional file 2, Figures 1S and 2S) with different cut-offs ranging from 8 to 13. The second best results were obtained with MH3_HMDB, while the metabolite identification method employed by HMDB NMR Search ranks third (0.98). When the cut-off is set to be equal with the number of metabolites in the mixture (13), the same method (MH1_HMDB) marginally outperforms the other four (see Additional file 1, Table 6S).

**Table 1:** Optimal cut-offs corresponding to maximal accuracies (in parentheses) for all 5 methods.

Data set	MH1_HMDB	MH1_MMCD	MH3_HMDB	MH3_MMCD	HMDB NMR Search
**SYN5_s**	8	4	8	1	1
	(**0.994**)	(0.971)	(0.992)	(0.969)	(0.985)
**SYN5_f**	9	1	5	1	1
	(**0.995**)	(0.973)	(0.991)	(0.973)	(0.985)
**SYN5_p**	13	14	7	7	1
	(**0.998**)	(0.984)	(0.993)	(0.982)	(0.987)
**EXP1**	1	1	1	1	1
	(0.984)	(0.969)	(0.984)	(0.969)	(**0.985**)
**EXP2**	1	2	1	1	1
	(0.986)	(0.973)	(**0.987**)	(0.973)	(0.985)

Data sets SYN5_s, SYN5_f and SYN5_p are synthetic mixtures and EXP1 and EXP2 are experimental mixtures. Each table cell contains the optimal K cut-off value (top K) and the corresponding maximal accuracy value (in parentheses).

For experimental data sets EXP1 and EXP2, HMDB NMR Search, MH1_HMDB and MH3_HMDB perform equally well with accuracies above 0.98, although in this case, the optimal cut-off is uniformly low (cut-off = 1) except for MH1_MMCD (cut-off = 2).

The AUC values for synthetic data sets (Table 2) are highest for the MH1_HMDB method (between 0.83 and 0.89), and they decrease to 0.51 and 0.48 for HMDB NMR Search and MH3_MMCD. The AUC values for experimental data sets drop dramatically below 0.5 (see Additional file 2, Figures 3S and 4S), given that the number of false positives is much higher due to unknown exact metabolite content (EXP1) and partially due to measurement of spectra using NMR spectrometers operating at much lower frequencies (270 MHz for EXP2) and thus lower resolution than the instruments used to acquire reference metabolite spectra (400-600 MHz).

**Table 2:** Area Under Curves (AUCs) for all 5 methods.

Data set	MH1_HMDB	MH1_MMCD	MH3_HMDB	MH3_MMCD	HMDB NMR Search
**SYN5_s**	**0.835**	0.636	0.653	0.484	0.516
**SYN5_f**	**0.839**	0.689	0.701	0.551	0
**SYN5_p**	**0.890**	0.849	0.663	0.589	0.739
**EXP1**	0.065	0.054	0.067	**0.137**	0.007
**EXP2**	0.170	0.171	0.221	0.153	**0.264**

Data sets SYN5_s, SYN5_f and SYN5_p are synthetic mixtures and EXP1 and EXP2 are experimental mixtures. Each table cell contains the Area Under Curve (AUC) value calculated using the trapezoid rule [37].

### Metabolite identification and results ranking

The practical utility of a metabolite identification method ultimately resides in its ability to identify as many metabolites as possible in a given mixture with as many valid matches as possible ranked closer to the top of the results list. In an ideal world, we would like to see the first 13 matches for data set SYN5_s as being the exact 13 metabolites from the mixture.

Here we investigate and compare the ability of the proposed methods (Tables 3 and 4) to find highly ranked correctly identified metabolites using two metrics, namely the percentage of correctly identified metabolites in top 100 results and the average rank of the identified metabolites. The first metric will quantify the solution while the second will qualify each result.

**Table 3:** Metabolite identification and results ranking for experimental mixtures.

Method	Sample	Total # metabolites	# Correctly identified (top 100)	%	AVG_Rank
**MH1_HMDB**	EXP1	4	2	**50.00**	58.50
**MH2_HMDB (0.01 ppm)**	EXP1	4	0	0.00	100.00
**MH3_HMDB**	EXP1	4	1	25.00	14.00
**MH1_MMCD**	EXP1	5	2	40.00	65.50
**MH2_MMCD**	EXP1	5	2	40.00	82.00
**MH3_MMCD**	EXP1	5	2	40.00	**12.50**
**HMDB NMR Search**	EXP1	4	1	25.00	91.00
**BMRB**	EXP1	5	1	20.00	26.00
**MMCD**	EXP1	5	2	40.00	20.50

**MH1_HMDB**	EXP2	5	3	60.00	27.33
**MH2_HMDB (0.01 ppm)**	EXP2	5	5	**100.00**	18.00
**MH3_HMDB**	EXP2	5	3	60.00	5.67
**MH1_MMCD**	EXP2	3	3	**100.00**	27.00
**MH2_MMCD**	EXP2	3	2	66.67	40.50
**MH3_MMCD**	EXP2	3	2	66.67	**2.00**
**HMDB NMR Search**	EXP2	5	4	80.00	15.50
**BMRB**	EXP2	5	1	20.00	22.00
**MMCD**	EXP2	5	1	20.00	54.00

BMRB and MMCD results were obtained using the following parameter settings: MMCD (method: NMR_based Search, H_tol = 0.05 ppm, Results to be shown: 100, Sample condition: D2O pD7.4 (273)), BMRB (method: NMR Peaks Query, Database to search: 1 H, H Range: 0.005).

**Table 4:** Metabolite identification and results ranking for synthetic mixtures.

Method	Sample	Total # metabolites	# Correctly identified (top 100)	%	AVG Rank
**MH1_HMDB**	SYN5_s	13	12	**92.31**	10.92
**MH2_HMDB (0.01 ppm)**	SYN5_s	13	12	**92.31**	34.50
**MH3_HMDB**	SYN5_s	13	9	69.23	**7.00**
**MH1_MMCD**	SYN5_s	13	11	84.62	26.09
**MH2_MMCD**	SYN5_s	13	10	76.92	18.20
**MH3_MMCD**	SYN5_s	13	7	53.85	11.43
**HMDB NMR Search**	SYN5_s	13	11	84.62	40.09
**BMRB**	SYN5_s	13	3	23.08	50.00
**MMCD**	SYN5_s	13	6	46.15	64.50

**MH1_HMDB**	SYN5_f	13	12	**92.31**	10.50
**MH2_HMDB (0.01 ppm)**	SYN5_f	13	12	**92.31**	34.25
**MH3_HMDB**	SYN5_f	13	10	76.92	**10.20**
**MH1_MMCD**	SYN5_f	13	11	84.62	19.82
**MH2_MMCD**	SYN5_f	13	11	84.62	28.64
**MH3_MMCD**	SYN5_f	13	8	61.54	11.88
**HMDB NMR Search**	SYN5_f	13	0	0.00	100.00
**BMRB**	SYN5_f	13	5	38.46	54.00
**MMCD**	SYN5_f	13	7	53.85	54.71

**MH1_HMDB**	SYN5_p	13	13	**100.00**	12.31
**MH2_HMDB (0.01 ppm)**	SYN5_p	13	13	**100.00**	19.31
**MH3_HMDB**	SYN5_p	13	9	69.23	**5.67**
**MH1_MMCD**	SYN5_p	13	12	92.31	9.42
**MH2_MMCD**	SYN5_p	13	12	92.31	20.08
**MH3_MMCD**	SYN5_p	13	8	61.54	5.75
**HMDB NMR Search**	SYN5_p	13	11	84.62	21.17
**BMRB**	SYN5_p	13	10	76.92	36.40
**MMCD**	SYN5_p	13	4	30.77	56.75

BMRB and MMCD results were obtained using the following parameter settings: MMCD (method: NMR_based Search, H_tol = 0.05 ppm, Results to be shown: 100, Sample condition: D2O pD7.4 (273)), BMRB (method: NMR Peaks Query, Database to search: 1 H, H Range: 0.005).

For experimental mixture EXP1, the highest percentage of metabolites out of top 100 was obtained with MH1_HMDB with an average rank of 58.5, while the best average rank (12.50) was obtained with MH3_MMCD. For this data set, the HMDB NMR Search identified only 25% (1 out of 4) of the metabolites with an average rank of 91. The NMR search tools employed by BMRB [36] and MMCD also identify up to 2 metabolites with lower average ranks compared to HMDB NMR Search.

For experimental mixture EXP2, the highest percentage of correctly identified metabolites (100%) was obtained with two methods, namely MH1_MMCD and MH2_HMDB with a chemical shift variation of ± 0.01 ppm, the latter having the lowest average ranking (18.00). Nevertheless, the best two average rankings were obtained with MH3_MMCD (2.00) and MH3_HMDB (5.67). HMDB NMR Search identified 4 out of 5 (80%) metabolites with an average ranking of 15.5, while the search tools of BMRB and MMCD identified only 1 out of 5 metabolites.

For synthetic mixtures (SYN5_s, SYN5_f, SYN5_p), the MH1_HMDB method consistently outperforms all the other methods, with match percentages of above 92% and average ranking below 13. For the same data sets, the HMDB NMR Search method correctly identifies up to 11 out of 13 metabolites (84.62%) for 2 out of three data sets with the best ranking of 21.17. For the third data set (SYN5_f), the same method is not able to identify any of the 13 metabolites, while the other methods seem to perform relatively well. When spectra of single metabolites are queried against the reference libraries (see Additional file 1, Table 1S), all of our methods clearly outperform HMDB NMR Search, BMRB, MMCD and Chenomx Profiler, both in terms of percentage of correctly identified metabolites and average ranking.

Overall, we notice that MH3_HMDB and MH3_MMCD produce the lowest average rankings regardless of the data set, at the expense of increasing the number of false negatives, while MH1_HMDB and MH1_MMCD typically identify a higher percentage of metabolites, but with slightly lower rankings.

### The effect of scoring functions on metabolite identification

**Figure 7:**
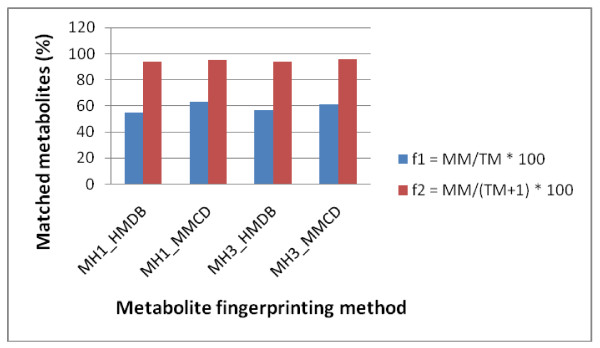
**Evaluation of MetaboHunter on individual metabolite spectra with methods that use different scoring functions**. Each individual metabolite spectrum was queried against the original reference libraries (HMDB and MMCD) using methods MH1 and MH3 that applied the scoring functions f1 (simple percentage calculations) and f2 (Equation 1) for ranking the metabolites. The top metabolite hit in MetaboHunter's output was reported as a match if it was identical with the query metabolite. The process was repeated for all metabolites in the reference libraries. MM = number of matched metabolites; TM = total number of metabolites.

Our modified scoring function (Equation 1) for metabolite identification was developed to insure that metabolites with larger numbers of peaks that have identical percentages of matched peaks as those with smaller number of peaks, should score higher. We prove that our novel scoring function was valid by performing an exhaustive search of single metabolites queried against the corresponding reference libraries (HMDB and MMCD). Figure 7 shows that MetaboHunter's metabolite identification accuracy averages 95% when the new scoring function (f2) is used, compared to 59% when the simpler percentage-based function used in HMDB NMR Search and other NMR metabolite fingerprinting tools is used.

## Conclusions

MetaboHunter provides an excellent tool for spectral assignment from ^1^H-NMR data with a novel and efficient scoring function and several different search methods. In the outlined examples MetaboHunter's search methods found metabolites included in the spectra with good accuracy. In many cases metabolites in question were obtained with the top, highest score, in the list, however, in some cases they were still listed but with lower scores.

Due to the large peak overlaps between different metabolites and the relatively large number of spectra present in the reference libraries, this can be expected and can only be dealt with either by doing multidimensional NMR or by utilizing a spectrum modelling from a more accurate and more focused metabolite database.

We expect that the accuracy of our methods will increase once metabolite spectral libraries obtained with higher resolution spectrometers (≥800 MHz) will become available. Such spectra typically contain more detailed peak resolutions, thus reducing the peak overlaps and increasing the ability of selection methods to choose among potential metabolite candidates present in a mixture.

We also acknowledge that, while MetaboHunter typically provides high quality results for synthetic and experimental input spectra of ^1^H-NMR metabolite mixtures with up to 13 components, more testing using spectra of mixtures with more components are necessary.

## Availability and Requirements

Project name: MetaboHunter

Project home page: http://www.nrcbioinformatics.ca/metabohunter

Application type: web server (browser independent)

## Methods

### Data collection, curation and pre-processing

The reference libraries information used in this study was collected from two publicly available resources, namely 876 metabolite peak lists (experimental) from the Human Metabolome Database, version 2.5 [28], and 448 metabolite peak lists from Madison Metabolomics Consortium Database [29]. The data was curated and re-formatted. The curation process consisted of manually adjusting the formatting inconsistencies (mostly found in the NMR Peaklist HMDB data files), that were further automatically processed and summarized into indexed tables for fast access. Specific metabolite data extracted from HMDB *metabocard *files was also re-organized and indexed to further facilitate its usage on the web server.

The data has been re-formatted as follows: (i) a set of peaks (ppm and height pairs) has been re-formatted with a 0.01 ppm precision and assigned to each metabolite. These pieces of information are subsequently used for metabolite identification and, (ii) each metabolite has been assigned a source of provenience (HMDB, MMCD), a type (drug, food additive, mammalian, microbial, plant, synthetic/industrial chemical), the pH of the sample in which it was measured (3.00 - 10.00), the solvent (water, CDCl3, CD3OD, 5% DMSO), and the frequency of the NMR machine (400 MHz, 500 MHz, 600 MHz).

**Figure 8:**
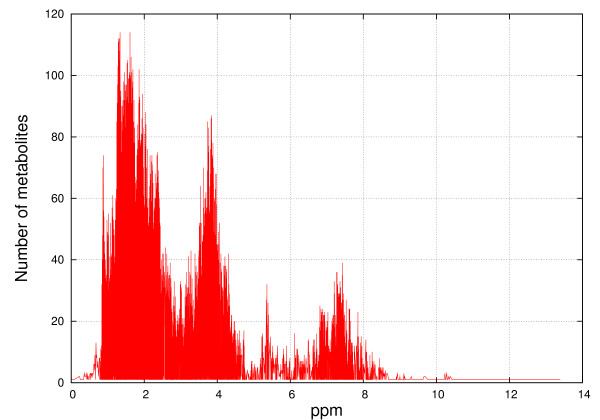
**Frequency of metabolites that share the same peak coordinate in the HMDB reference library**. The x-axis contains the locations of the peaks (ppm) and the y-axis marks the number of metabolites that have peaks at given locations.

**Figure 9:**
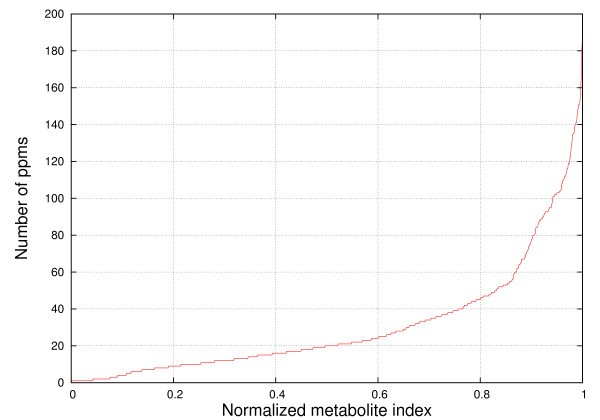
**Distribution of the number of peak coordinates per metabolite in the HMDB reference library**. The number of peak coordinates for HMDB metabolites varies between 1 and 181.

**Figure 10:**
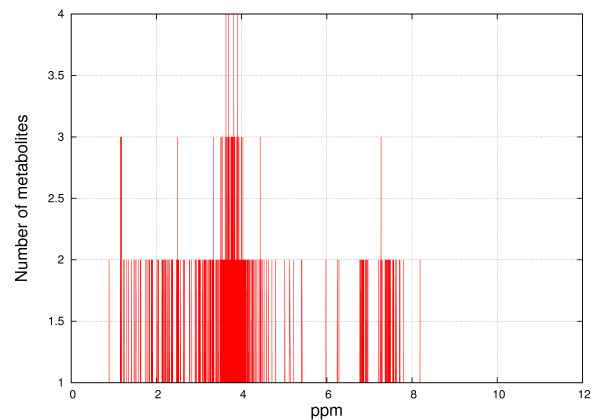
**Frequency of metabolites that share the same peak coordinate in the MMCD reference library**. The x-axis contains the locations of the peaks (ppm) and the y-axis marks the number of metabolites that have peaks at given locations.

**Figure 11:**
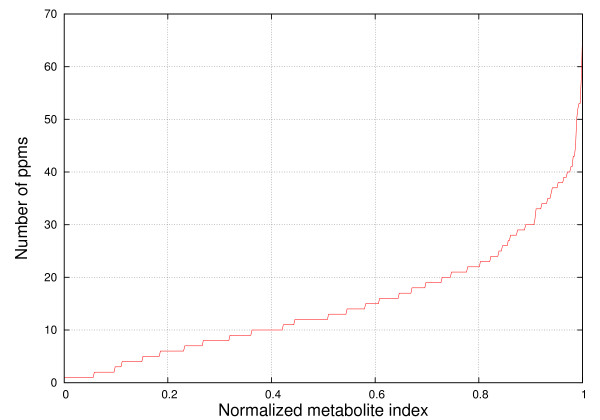
**Distribution of the number of peak coordinates per metabolite in the MMCD reference library**. The number of peak coordinates for HMDB metabolites varies between 1 and 66.

Figures 8, 9, 10 and 11 show the frequencies of metabolites from HMDB and MMCD data that share the same peak coordinates and the distribution of the number of peaks for all metabolites. The number of metabolites sharing the same peak coordinate is much higher for HMDB reference metabolites than for the ones in MMCD, while the number of metabolites in HMDB is roughly only about two times higher than MMCD.

### Datasets

The datasets used in this study consist of both, synthetic and experimental data. The synthetic data can be categorized as: ^1^H-NMR spectra (_s), list of peaks extracted externally (with MNova) from spectra (_f) and mixtures of peaks obtained directly from HMDB peak lists (_p). The following **synthetic data sets **are considered:

### Single metabolites

- **SYN1_s**: Acetylcholine HMDB spectrum,

- **SYN1_f**: Acetylcholine HMDB spectral peaks,

- **SYN1_p**: Acetylcholine HMDB database peaks,

- **SYN2_s**: 17a-Estradiol HMDB spectrum,

- **SYN2_f**: 17a-Estradiol HMDB spectral peaks,

- **SYN2_p**: 17a-Estradiol HMDB database peaks,

- **SYN3_s**: Cholesterol HMDB spectrum,

- **SYN3_f**: Cholesterol HMDB spectral peaks,

- **SYN3_p**: Cholesterol HMDB database peaks,

- **SYN4_s**: D-Glucose HMDB spectrum,

- **SYN4_f**: D-Glucose HMDB spectral peaks,

- **SYN4_p**: D-Glucose HMDB database peaks.

### Multiple metabolites

- **SYN5_s**: Mixture of 13 metabolites spectrum,

- **SYN5_f**: Mixture of FIDs for 13 metabolites from HMDB spectral peaks,

- **SYN5_p**: Mixture of 13 metabolites HMDB peaks.

The 13 metabolites included in SYN5 are: Choline, Glutathione, L-Alanine, L-Glutamic Acid, L-Glutamine, L-Leucine, L-Valine, L-Asparagine, L-Isoleucine, L-Lactic acid, L-Proline, Succinic acid and Taurine.

The **experimental data **includes:

- **EXP1**: ^1^H-NMR spectrum of a spiked-in urine sample from a healthy individual obtained from Zheng *et al. *[21]. The spectrum was measured on a 500 MHz Bruker Avance NMR spectrometer. The five spiked-in metabolites in this sample are: Taurine, Hippuric acid, Nicotinate, Malic acid and Oxoglutaric acid. The remaining metabolites in the mixture are not known.

- **EXP2**: An experimental mixture of 5 metabolites measured on a 270 MHz Jeol spectrometer at 25°C. Mixture was prepared under N_2_ atmosphere, in 99.8% D_2_O with reference substance (3-(trimethylsilyl)propionic-2,2,3,3-d4 acid, sodium salt, 98% atom% D, TMSP, at 0.6 mM). The five metabolites included in this sample are: Creatine, D-Glucose, Citric acid, Phosphocholine and Accetylcholine.

### Peak identification and noise removal

**Figure 12:**
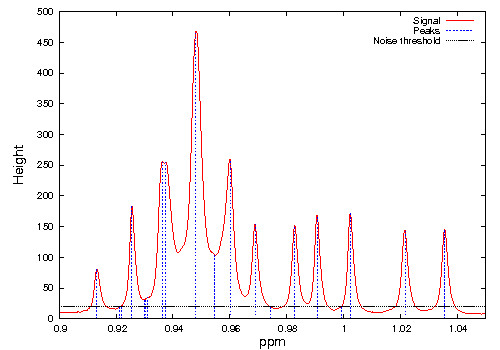
**De-noising and peaks identification in an NMR spectrum**. The spectrum data points lie on the red curve while the vertical dotted blue segments represent peak locations whose heights are above the noise threshold (horizontal black line).

The user input for MetaboHunter consists of a complete spectrum in ASCII format structured on two columns, first one representing the spectral point position (ppm) and the second column representing the corresponding intensity of the signal. Based on a user-provided threshold for noise level, i.e. the minimum intensity level above which peaks should be considered, the data is pre-processed so that data points with intensity below the threshold are removed and the peaks are automatically identified (see Figure 12).

### Exact and variable peaks matching

In MetaboHunter, a metabolite is considered to be present if at least one of its peaks matches a peak in the test sample and the computed significance score is greater than a user-set threshold (default is 0.5).

While the significance score proposed in [6] computes the ratio between the number of matched peaks and the total number of peaks for each metabolite, here we propose a metabolite significance score that is able to differentiate between matches of metabolites with low number of peaks as opposed to those with greater number of peaks. The significance score is computed as the ratio between: (i) the number of matched metabolite peaks and (ii) one plus the total number of metabolite peaks (Equation 1). For example, one metabolite with 2 out of 4 peaks matching a sample gets a score of 0.4, while another metabolite with 5 out of 10 peaks matching the same sample will get a higher score of 0.45, thus being ranked higher.

Equation 1. A novel scoring function used by all three methods implemented in MetaboHunter.

$$significance\;score=\frac{total\;number\;of\;matched\;peaks}{1+total\;number\;of\;peaks}$$

Exact matches for metabolite peak locations can be considered when chemical shifts of metabolites in the sample are assumed to be the same as in the reference database. Thus we provide an implementation of this approach based on the significance score described above. Methods MH2 and MH3 allow variations in chemical shifts and take as input a user-set parameter τ representing the maximal range allowed. Each metabolite peak is matched against all sample peaks *p *that fall within the interval [*p*-*τ*, *p*+*τ*] and the corresponding significance score is calculated and reported.

### Metabolite fingerprinting methods

For the detection of metabolites using ^1^H-NMR spectra of sample mixtures, we use three methods.

The first method (**MH1**) relies on exact matching of peaks from the mixture with reference metabolite spectrum peaks collected and carefully curated from two publicly available databases (HMDB and MMCD). The method starts by computing and sorting initial scores using Equation 1 based on peak matches between the test spectrum and all the library spectra. Next, the matched metabolites are then screened based on the desired user features, such as metabolite type, sample pH, solvent, and NMR frequency and only those with scores larger than the desired confidence threshold are displayed. The method is more accurate if the spectral measurements of the mixture and of the metabolites in the database are performed under the same NMR and sample conditions, otherwise, peak position changes can lead to false metabolite identifications.

#### Pseudo-code for MH1

*Input: *A spectrum (or set of peaks) *S *of a mixture of metabolites *M = {m*_*1*_, *m*_*2*_*,*..., *m*_*n*_}, a library *L *with *k *metabolites *{l*_*1*_, *l*_*2*_*,*..., *l*_*k*_*}, *a noise threshold *η*, a ranking threshold *r, *and a set of metabolite features *F.*

*Output: *A set of metabolites predicted to be in the mixture.

Method:

1. Pre-process *S: *remove all peaks in *S, *whose amplitudes are below *η*.

2. **for ***i = *1*:k*

3.    Compute significance score *S*_*i*_ for each library metabolite *l*_*i*_ without horizontal peak drift

4.    Output metabolite *l*_*i*_ if *S*_*i*_ ≥ *r *and *l*_*i*_ features obey *F*.

5. **end for**

To partially alleviate this inconvenience, a second method (**MH2**) was proposed that allows inexact one dimensional match of peak chemical shifts (measured in parts per million - ppm) via horizontal peak drifts by a not constrained user-defined margin *(e.g. *a small chemical shift variation of ± 0.005 ppm or no variation at all). This feature offers more flexibility than HMDB NMR Search [28] and MetaboMiner [6] towards counter-acting the effects of measurement and pre-processing variability introduced by factors such as: different instruments, pH values and solvents. In this case, the number of matches typically increases with increased tolerance level around a given peak location, thus increasing also the number of false positives. The main difference in functionality between MH2 and MH1 resides in an adjusted matching criterion for computing the scores obtained with Equation 1. Two peaks are considered to match if their shift position is within the given "shift tolerance" parameter value, and thus the scores obtained with MH2 are typically higher than those computed with MH1.

#### Pseudo-code for MH2

*Input: *A spectrum (or set of peaks) *S *of a mixture of metabolites *M *= {*m*_*1*_,*m*_*2*_,...,*m*_*n*_}, a library *L *with *k *metabolites {*l*_*1*_,*l*_*2*_,...,*l*_*k*_}, a noise threshold *η*, a ranking threshold *r, *a user-set peak drift margin *δ*, and a set of metabolite features *F*.

*Output: *A set of metabolites predicted to be in the mixture.

Method:

1. Pre-process *S*: remove all peaks in *S*, whose amplitudes are below *η*.

2. **for ***i *= 1:*k*

3.    Compute significance score *S*_*i*_ for each library metabolite *l*_*i*_ with *δ *horizontal peak drift

4.    Output metabolite *l*_*i*_ if *S*_*i*_ ≥ *r *and *l*_*i*_ features obey *F*.

5. **end for**

The third method (**MH3**) consists of a greedy selection approach that enforces mutual exclusion of peaks via an iterative coordinate removal for each selected peak. In the same fashion as for MH1, MH3 starts by computing and sorting initial scores using Equation 1 based on peak matches between the test spectrum and all the library spectra. Next, it proceeds in an iterative manner by selecting the library metabolite with the highest score and highest number of peaks (in case of a tie) and then removing the corresponding peaks for the selected metabolite from the remaining pool of unassigned peaks. The method stops when no spectral peaks remain to be assigned. This approach favours the early selection of metabolites with higher scores and higher number of peaks, thus decreasing the number of false positives (through iterative removal of remaining peak coordinates) at the cost of increasing the number of false negatives. False negatives typically happen in this method for metabolites in the mixture with a high degree of overlapping peak coordinates. MH3 also allows a user-defined shift tolerance around peak locations (not evaluated here and thus experimental). We also note that MH3 could be further improved so that to better mimic the processing of human experts, if after iteratively removing the corresponding spectrum of one metabolite, the remaining spectrum will be further adjusted to take into account the effect of the removed components on the mixture spectrum. Nevertheless, supporting information that could help with this step is not available at this time.

#### Pseudo-code for MH3

*Input: *A spectrum (or set of peaks) *S *of a mixture of metabolites *M *= {*m*_*1*_,*m*_*2*_,...,*m*_*n*_}, a library *L *with *k *metabolites {*l*_*1*_,*l*_*2*_,...,*l*_*k*_}, a noise threshold *η*, a ranking threshold *r, *a user-set peak drift margin δ, and a set of metabolite features *F*.

*Output: *A set of metabolites predicted to be in the mixture.

Method:

1. Pre-process *S*: remove all peaks in *S*, whose amplitudes are below *η*.

2. **for ***i *= 1:*k*

3.    Compute significance score *S*_*i*_ for each library metabolite *l*_*i*_ with δ horizontal peak drift

4. **end for**

5. Sort metabolites in reverse order based on their significance scores

6. **while **(number of peaks in *S *≠ 0)

7.    **for ***i *= 1:*k*

8.       Select metabolite *l*_*i*_ with highest significance score with/without *δ *horizontal peak drift

9.       Output metabolite *l*_*i*_ if *S*_*i*_ ≥ *r *and *l*_*i*_ features obey *F*.

10.       Remove all peaks from *S *corresponding to metabolite *l*_*i*_

11.    **end for**

12.    Re-sort metabolites in reverse order based on their updated significance scores

13. **end while**

Given the two reference libraries used as support for the proposed methods, we append the suffixes "_HMDB" and "_MMCD" to the corresponding method names.

### Results evaluation

**Figure 13:**
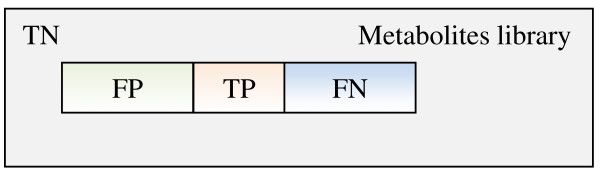
**Confusion matrix**. The number of true negatives (TN) is calculated as the difference between the number of metabolites in the reference library minus the total number of true positives (TP), false positives (FP) and false negatives (FN).

Similarly to the methodology described in [6], we perform our analysis by defining the confusion matrix (Figure 13), which includes:

-**True Positives (TP)**: The number of metabolites correctly identified as being present in the mixture.

-**True Negatives (TN)**: The number of metabolites correctly identified as not being present in the mixture. This includes the total number of metabolites in the library minus TP, FP and FN.

-**False Positives (FP)**: The number of metabolites incorrectly identified as being part of the mixture.

-**False Negatives (FN)**: The number of metabolites being present in the mixture but unidentified by the method.

Each of the measures defined above requires a cut-off threshold defined as the first N metabolites predicted to be in the mixture. The size of the metabolite libraries, upon which the TN is defined, is 876 for our HMDB library, 448 for our MMCD library and 916 for the online HMDB NMR Search DB.

The following statistics are used for performance evaluation of the peak identification methods proposed in this article:

$$\begin{array}{c} \text{Specificity}:\;\frac{TN}{TN+FP}\\ \text{Sensitivity}:\;\frac{TP}{TP+FN}\\ \text{Accuracy}:\;\;\frac{TP+TN}{TP+TN+FP+FN} \end{array}$$

Unless otherwise specified, all results obtained with MetaboHunter and reported in this article were obtained using water as solvent and zero shift tolerance.

### Run times and computational settings

The absolute CPU time for each method was measured on a PC with one 2.8 GHz Intel Pentium 4 CPU, 512 KB cache and 1 GB RAM running Mandriva Linux release 2010.2 (kernel 2.6.33.7). The obtained run time values for MH3_HMDB range from 2.1 seconds for input peak list files with 5 metabolites to 7 seconds for files with 100 metabolites, while for MH1_HMDB the run times range from 3.4 seconds to 5.7 seconds for the same input files. The run time estimates were averaged over 10 runs for each method and for each input peak list file. When MMCD is used as reference library, the average run times decrease in average with 3.5 seconds for input files with peak lists corresponding to mixtures of 100 metabolites. The decrease in run time is caused by the reduced size of the MMCD reference library (448 spectra) compared to HMDB (867 spectra).

Throughout the paper we used default settings for all publicly available software, such as HMDB NMR Search, BMRB, and Chenomx Profiler, when not specified otherwise. In the case of the free evaluation copy of Chenomx Profiler, we could only test it on data sets where FID files were accessible (SYN1-SYN4), since the software does not accept lists of peaks as input.

### Web user interface

**Figure 14:**
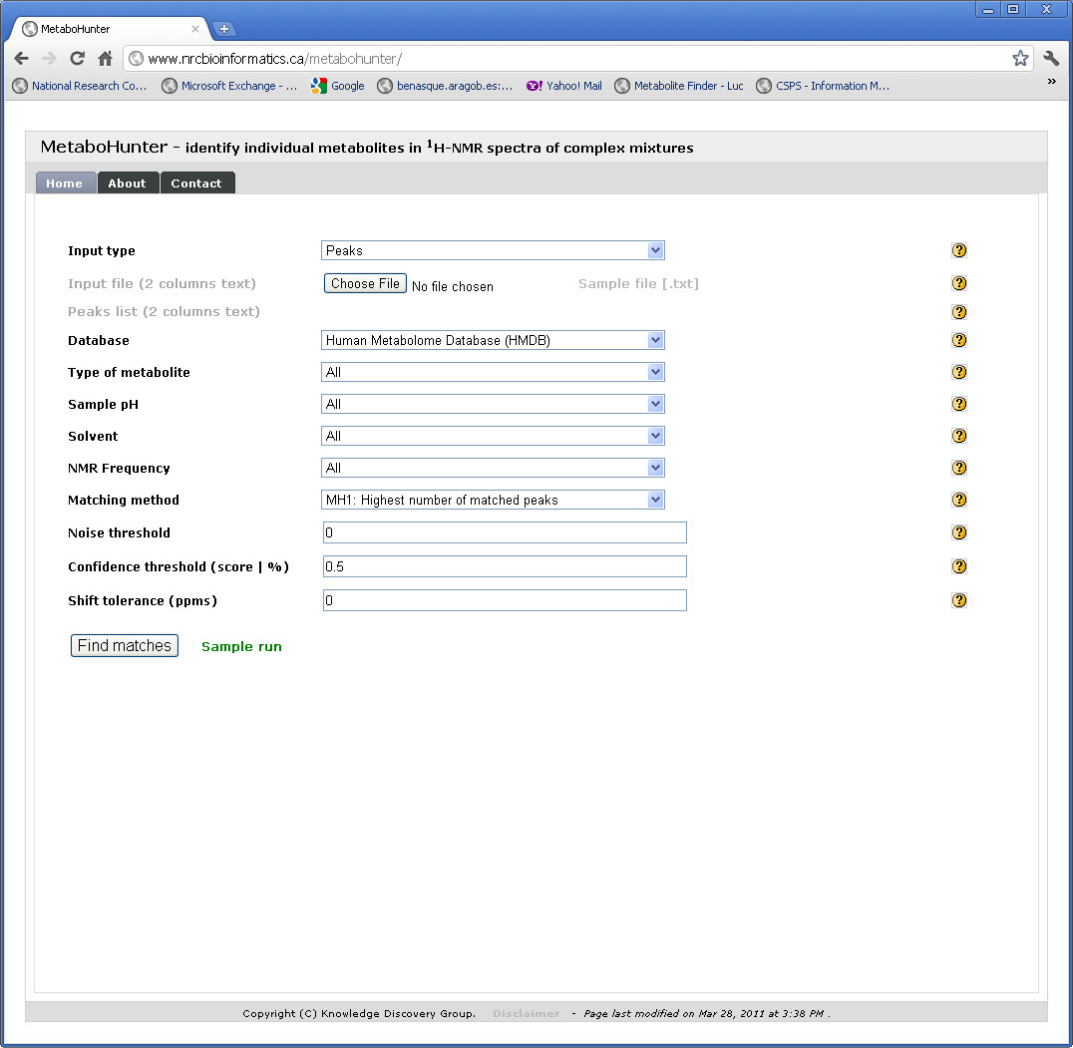
**MetaboHunter screenshot for the Processing View**. The figure shows the Processing View for MetaboHunter, which includes drop-down selection lists for input type, reference library (database), metabolite type, pH, solvent, NMR frequency, matching method, noise threshold, confidence threshold and shift tolerance.

MetaboHunter was implemented in Perl and its graphical user interface was developed in PHP, thus being browser independent. There are 4 functional views in MetaboHunter: (i) a Processing View, (ii) a Search Results View, (iii) a Plot View and, (iv) a Peaks Hit Map view. When the user accesses MetaboHunter web site, the Processing View (Figure 14) is the default view. The user can provide its input as a 2 column full spectrum or list of peaks, which can be uploaded as a text file or be copied and pasted in a text box.

**Figure 15:**
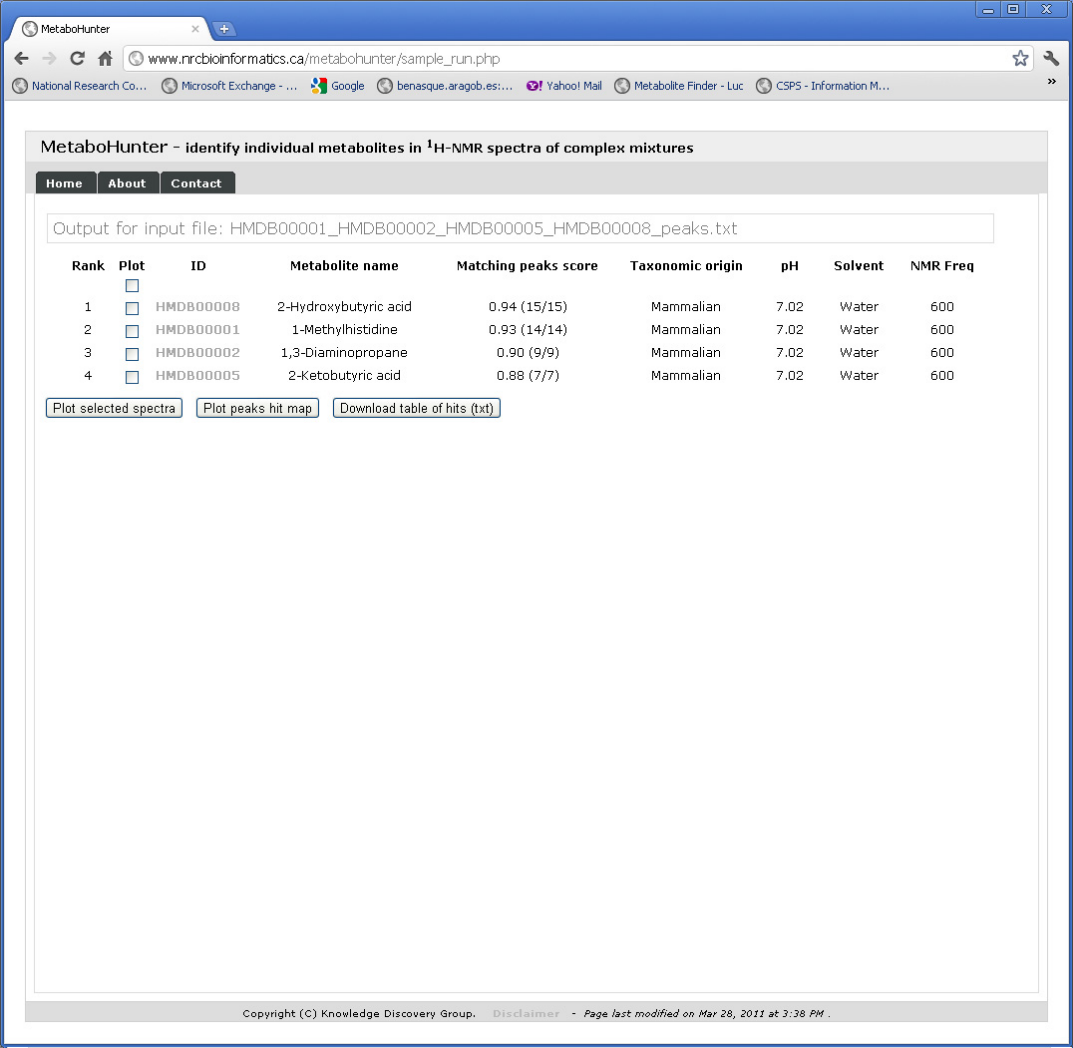
**MetaboHunter screenshot for the Search Results View**. The figure depicts MetaboHunter's Search Results View, which consists of metabolite ranking, plot selection field, metabolite ID, metabolite name, matching score and ratio of identified versus total number of peaks, origin of reference metabolite, pH, solvent and the experimental NMR frequency. Three action buttons are placed at the bottom of the list of results, which allow users to further select, download and explore the identified metabolites using graphical means.

MetaboHunter has a comprehensive output for identified metabolites in input spectra or lists of peaks. The output is depicted in the Search Results View (Figure 15), which includes a ranked list of metabolite IDs, names and taxonomic origin, direct links to their descriptive original web pages, scoring information, and links to spectral plots and peaks hit map visualization of selected results. The results displayed in the Search Results View can be downloaded as text.

**Figure 16:**
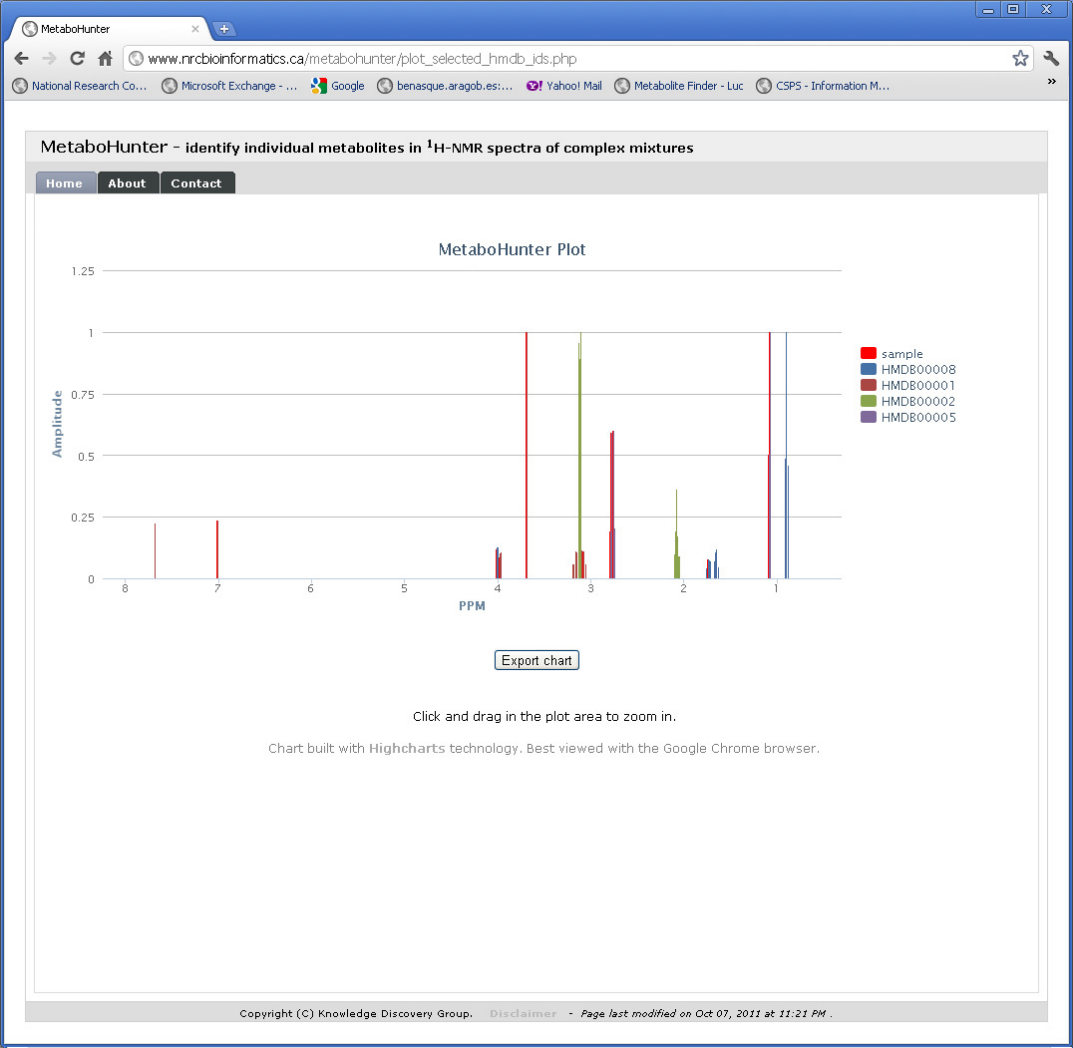
**MetaboHunter screenshot for the Plot View**. The figure shows MetaboHunter's Plot View, which lists on the left the selected metabolites, while the plot on the right shows the location of the selected metabolite peaks with respect to the sample peaks. The "Export chart" button at the bottom of the plot allows users to save the plot as a PDF file.

**Figure 17:**
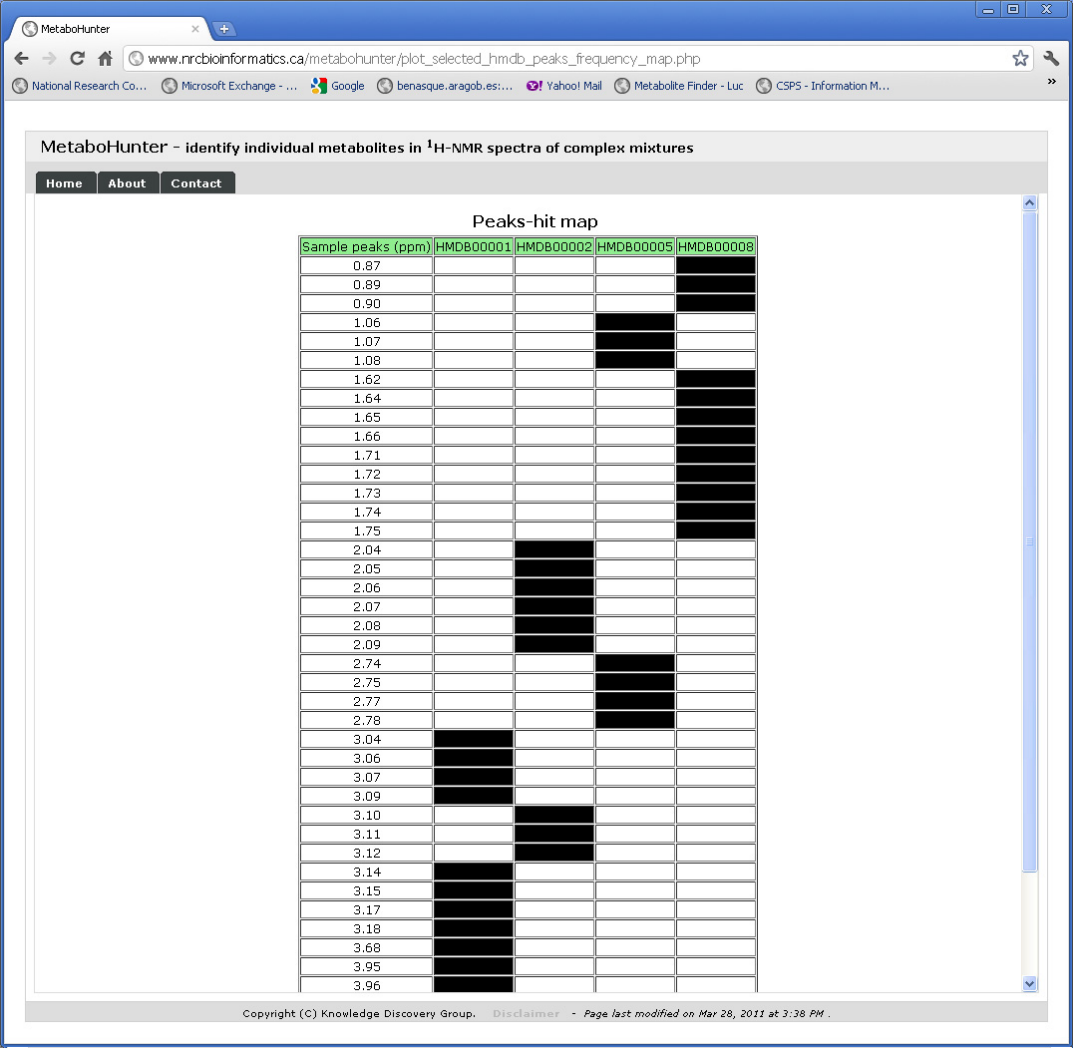
**MetaboHunter screenshot for the Peaks Hit Map View**. The figure shows MetaboHunter's Peaks Hit Map View, which displays in a tabular fashion the identity of the identified metabolite peaks relative to the location (ppm) of all the peaks in the sample.

The Plot and Peaks Hit Map Views depicted in Figures 16 and 17 permit users to visualize the overlap between selected peaks corresponding to specific metabolites and the input data, as well as, the shared and disjoint peak locations for selected metabolites in the output. The plots are automatically generated using the Highcharts interactive JavaScript library and can be exported as PDF files.

## Authors' contributions

MCC and DT conceived, designed and coordinated the study and helped to draft the manuscript. SL, LB and DT participated in the programming of the application and methods. AC and MCC performed the experimental measurements. MCC and DT participated in the evaluation of the methods. All authors read and approved the final manuscript.

## Supplementary Material

Additional file 1 **Supplemental information**. The file contains tables with detailed performance results for all experimental (EXP1, EXP2) and synthetic data sets (SYN1 - SYN5). Click here for file

Additional file 2 **Supplemental information**. The file contains additional pictures representing ROC curves and accuracy variability depending on result list cut-offs for experimental data sets EXP1 and EXP2 and for synthetic data sets SYN5_s, SYN5_f and SYN5_p. Click here for file
