# Supplementary material for: MetaboHunter: an automatic approach for identification of metabolites from 1H-NMR spectra of complex mixtures
Source: BMC Bioinformatics. 2011 Oct 14;12:400. doi: 10.1186/1471-2105-12-400 (PMC3213069; doi:10.1186/1471-2105-12-400)
Supplement: Additional file 1 — Supplemental information. The file contains tables with detailed performance results for all experimental (EXP1, EXP2) and synthetic data sets (SYN1 - SYN5). [file 1471-2105-12-400-S1.PDF]

Supplemental information – figures

Figure 1S. ROC curves for the MH1_HMDB, MH1_MMCD, MH3_HMDB, MH3_MMCD and HMDB NMR Search methods applied to synthetic data: SYN5_s, SYN5_f and SYN5_p. The cut-off was varied from 1 to 100.

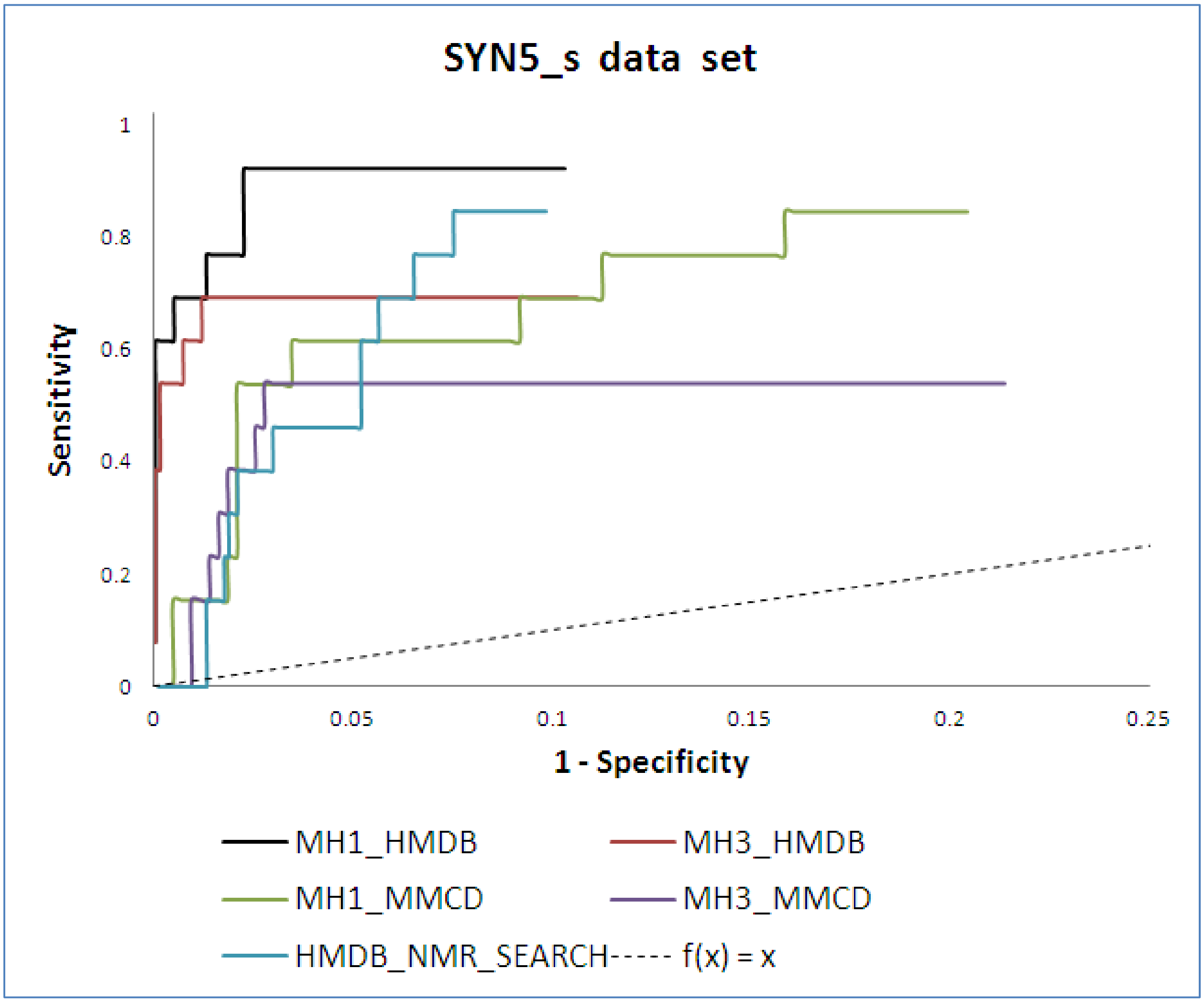

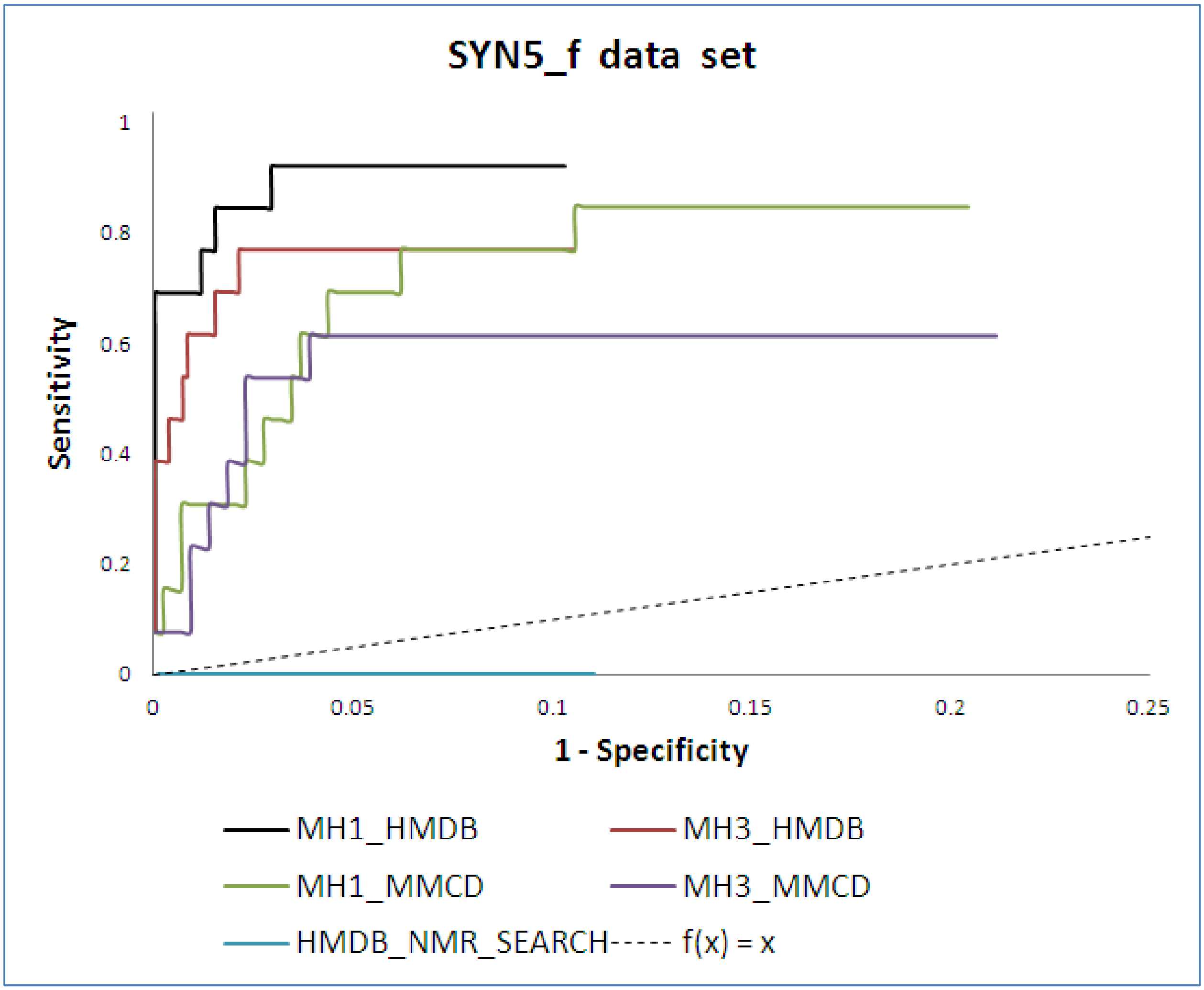
SYN5_f data set
Sensitivity
1
0.8
0.6
0.4
0.2
0
0
0.05
0.1
0.15
0.2
0.25
1 - Specificity
MH1_HMDB
MH3_HMDB
MH1_MMCD
MH3_MMCD
HMDB_NMR_SEARCH
f(x) = x

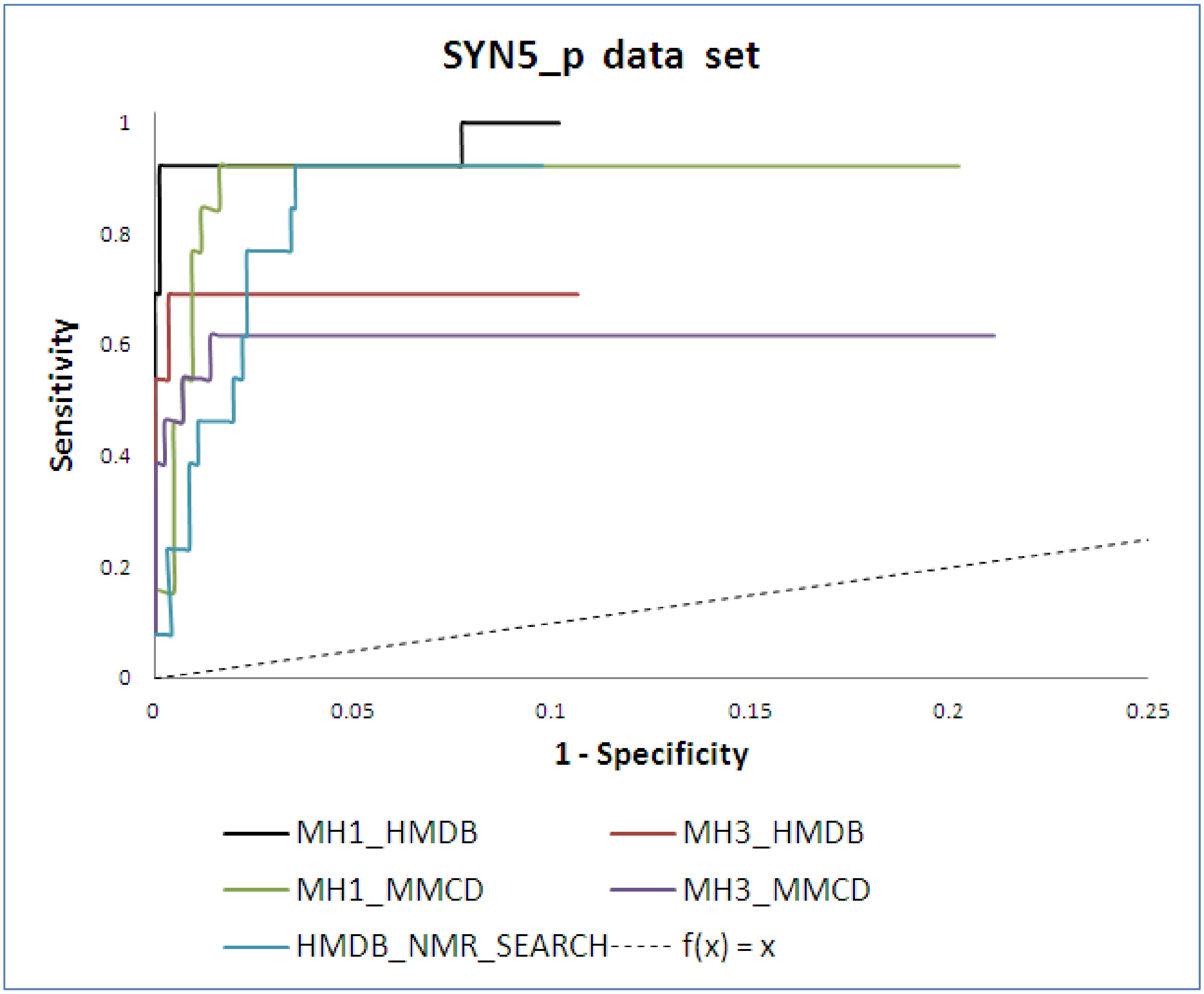
SYN5_p data set
Sensitivity
1
0.8
0.6
0.4
0.2
0
0
0.05
0.1
0.15
0.2
0.25
1 - Specificity
MH1_HMDB
MH3_HMDB
MH1_MMCD
MH3_MMCD
HMDB_NMR_SEARCH
f(x) = x

Figure 2S. Accuracy variation with cut-off for the MH1_HMDB, MH1_MMCD, MH3_HMDB, MH3_MMCD and HMDB NMR Search methods applied to synthetic data: SYN5_s, SYN5_f and SYN5_p. The cut-off was varied from 1 to 100.

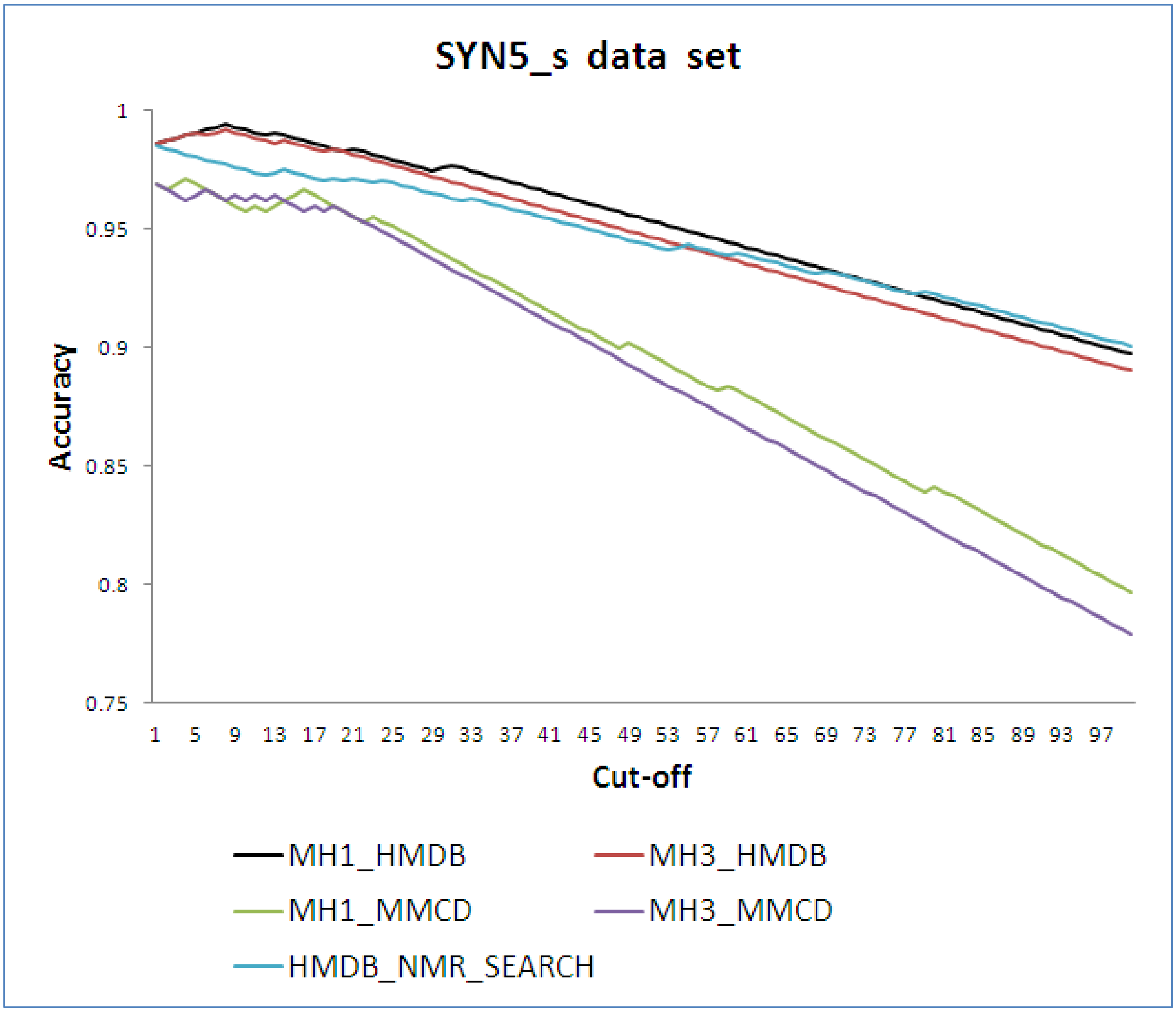

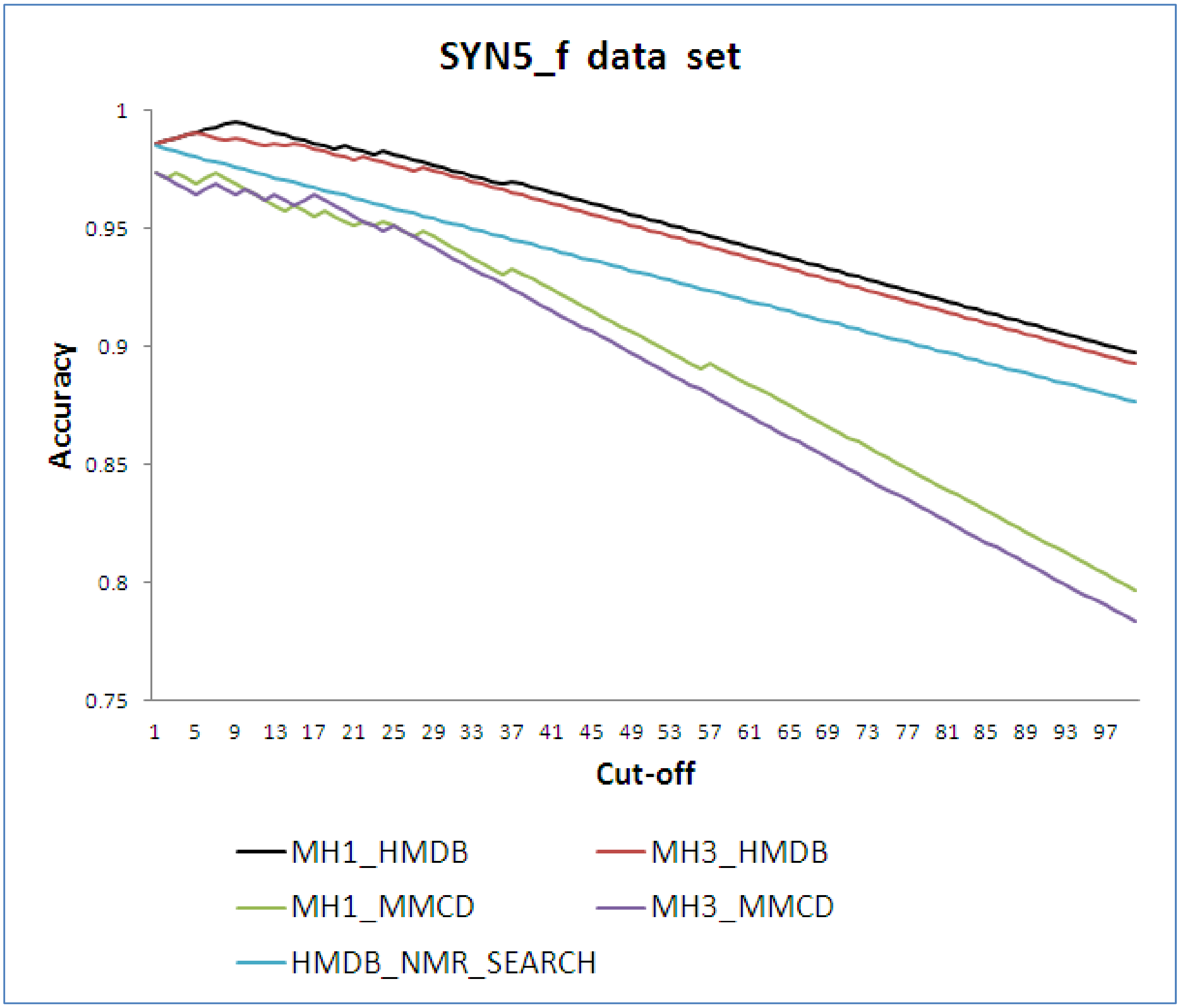
SYN5_f data set
Accuracy
1
0.95
0.9
0.85
0.8
0.75
1 5 9 13 17 21 25 29 33 37 41 45 49 53 57 61 65 69 73 77 81 85 89 93 97
Cut-off
MH1_HMDB
MH3_HMDB
MH1_MMCD
MH3_MMCD
HMDB_NMR_SEARCH

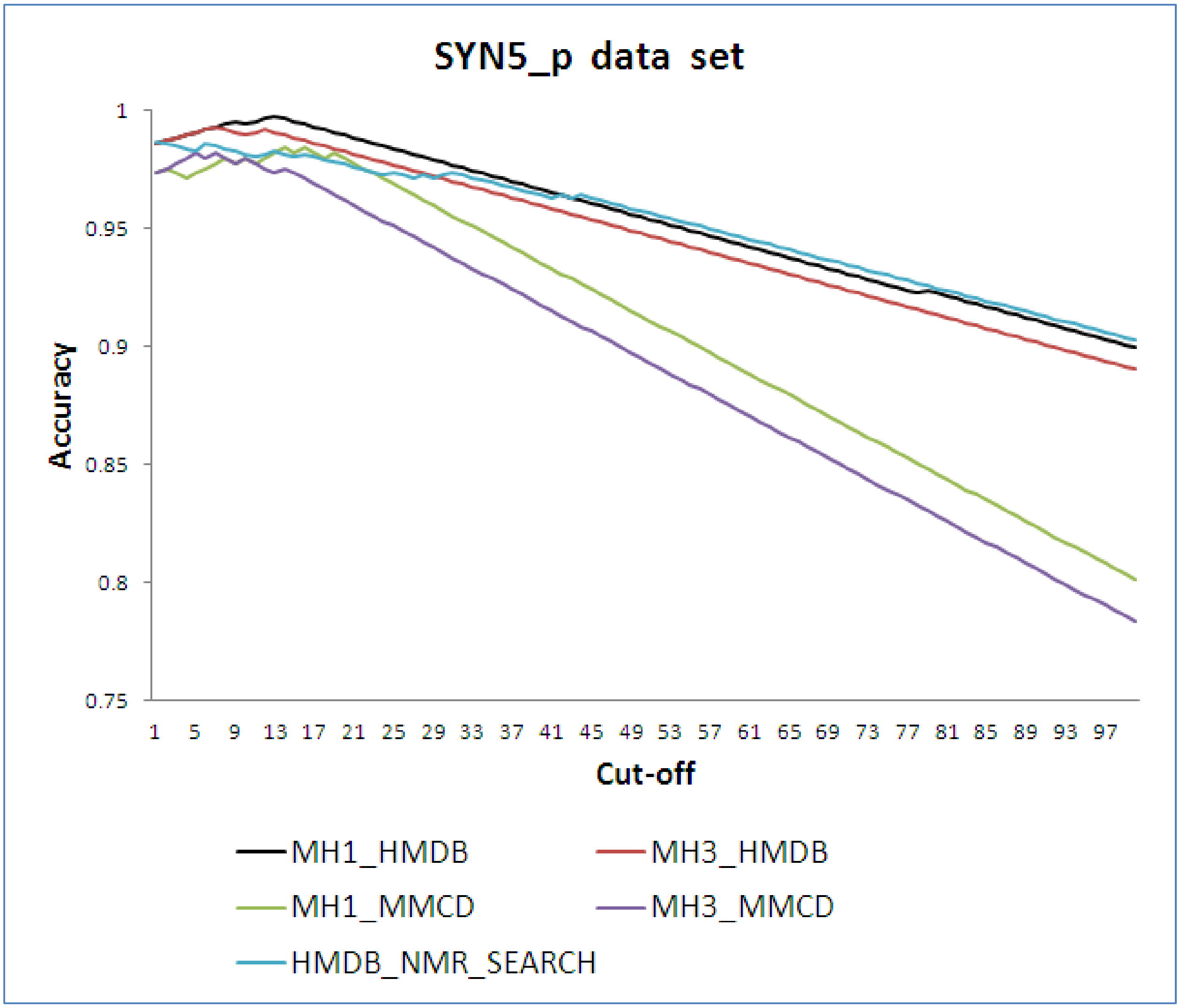
SYN5_p data set
Accuracy
1
0.95
0.9
0.85
0.8
0.75
1 5 9 13 17 21 25 29 33 37 41 45 49 53 57 61 65 69 73 77 81 85 89 93 97
Cut-off
MH1_HMDB
MH3_HMDB
MH1_MMCD
MH3_MMCD
HMDB_NMR_SEARCH

Figure 3S. ROC curves for the MH1_HMDB, MH1_MMCD, MH3_HMDB, MH3_MMCD and HMDB NMR Search methods applied to experimental data: EXP1 and EXP2. The cut-off was varied from 1 to 100.

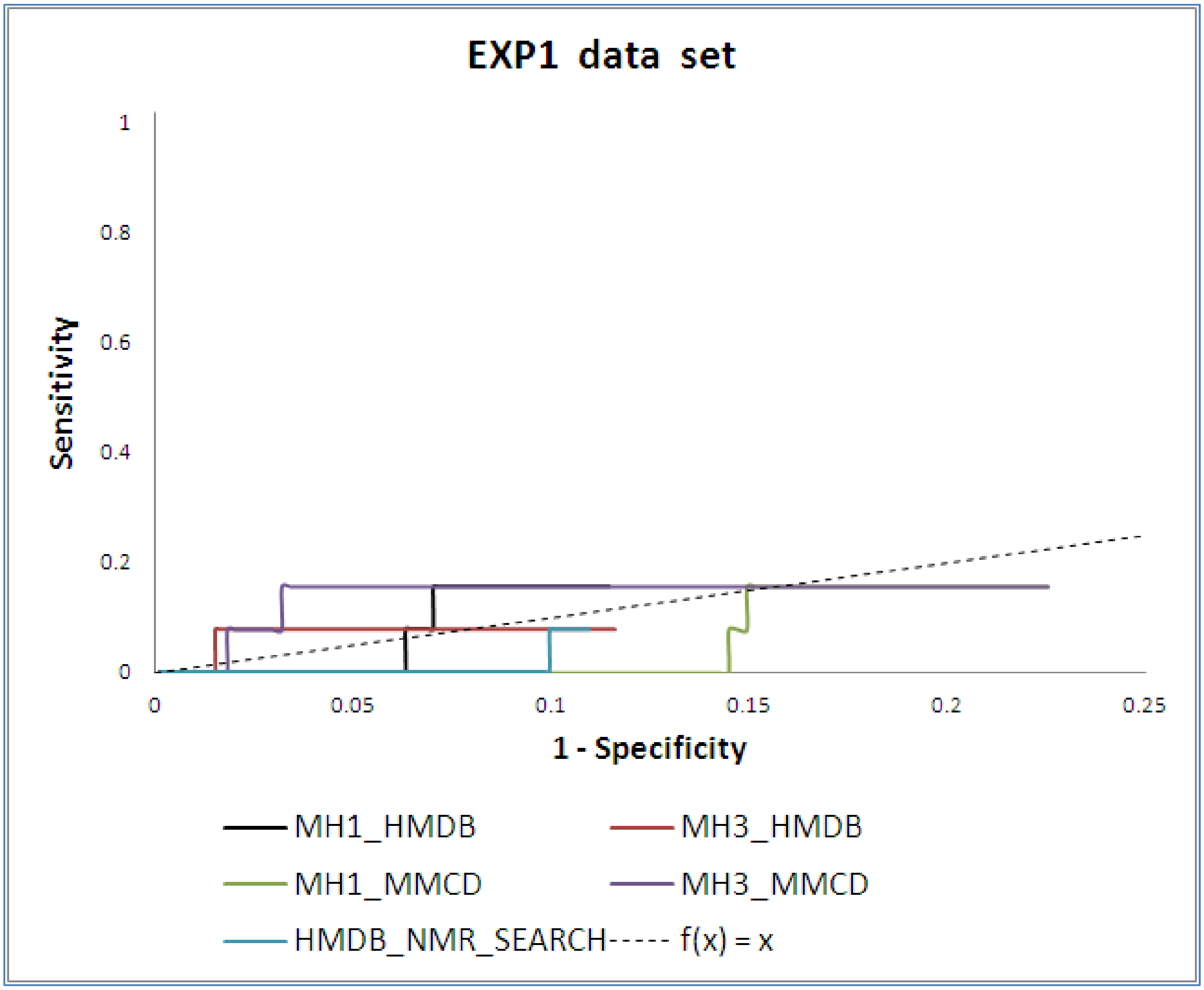

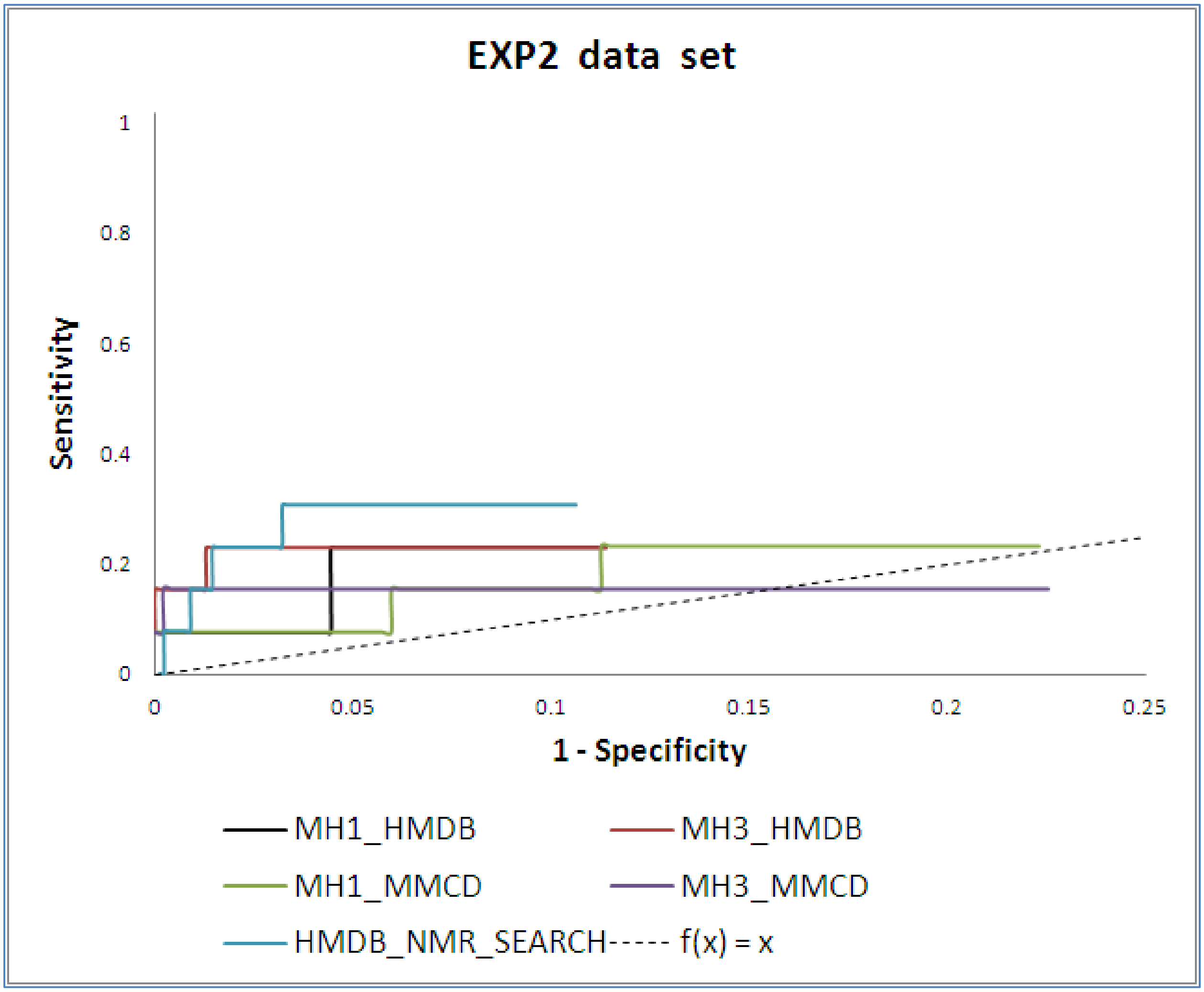
EXP2 data set
Sensitivity
1
0.8
0.6
0.4
0.2
0
0
0.05
0.1
0.15
0.2
0.25
1 - Specificity
MH1_HMDB
MH3_HMDB
MH1_MMCD
MH3_MMCD
HMDB_NMR_SEARCH
f(x) = x

Figure 4S. Accuracy variation with cut-off for the MH1_HMDB, MH1_MMCD, MH3_HMDB, MH3_MMCD and HMDB NMR Search methods applied to experimental data: EXP1 and EXP2. The cut-off was varied from 1 to 100.

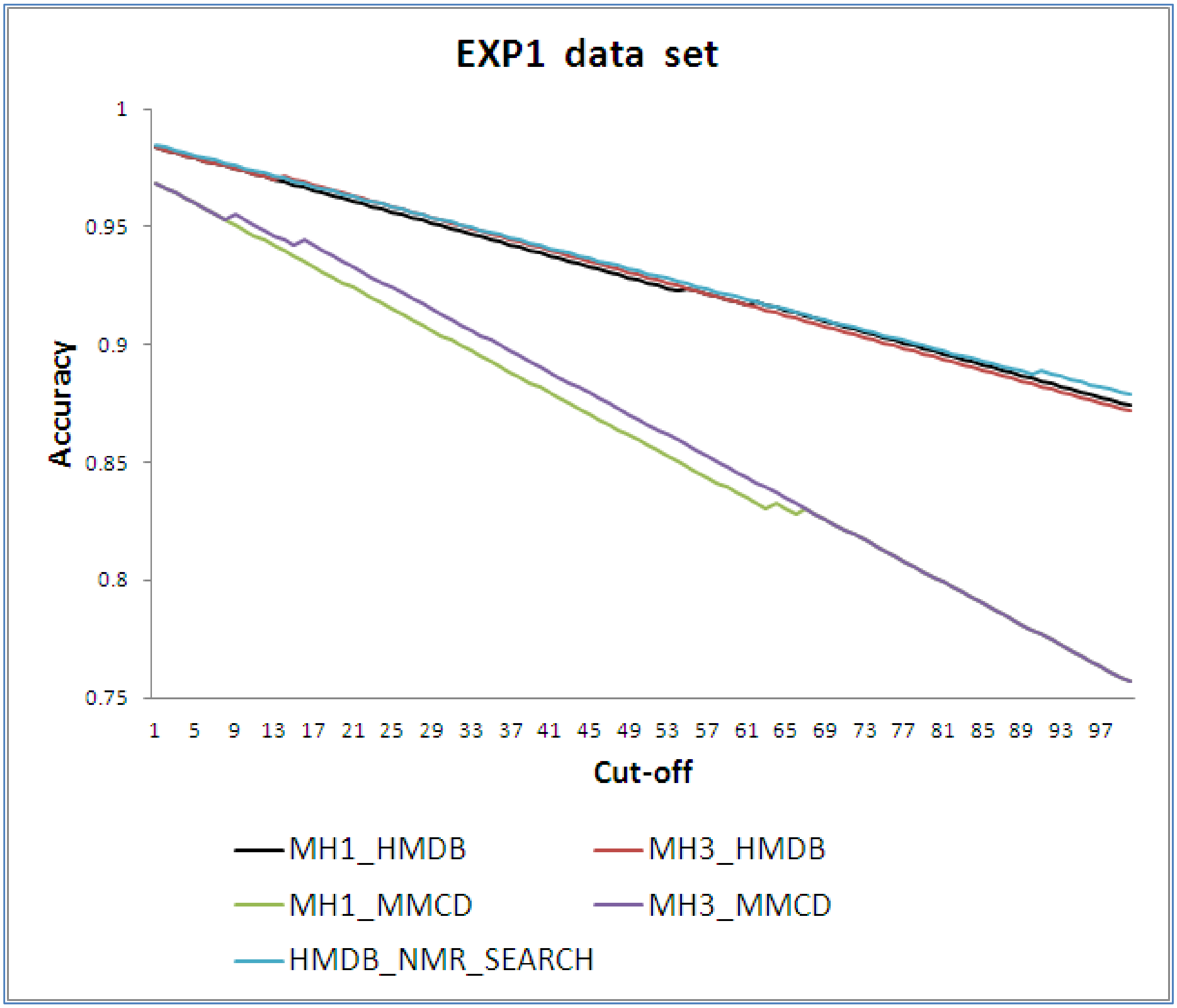

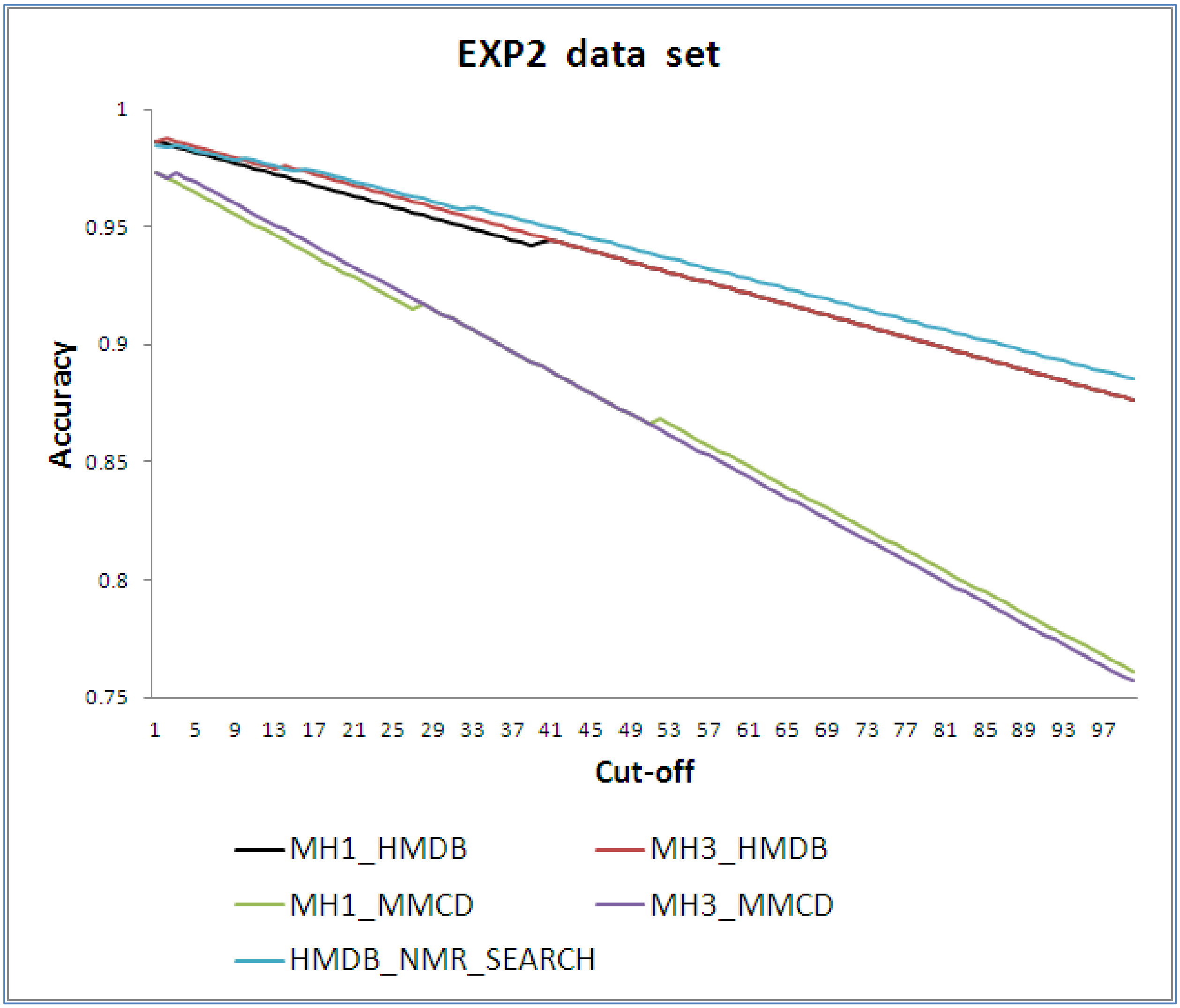
EXP2 data set
Accuracy
1
0.95
0.9
0.85
0.8
0.75
1 5 9 13 17 21 25 29 33 37 41 45 49 53 57 61 65 69 73 77 81 85 89 93 97
Cut-off
MH1_HMDB
MH3_HMDB
MH1_MMCD
MH3_MMCD
HMDB_NMR_SEARCH
