# Supplementary material for: MetaboHunter: an automatic approach for identification of metabolites from 1H-NMR spectra of complex mixtures
Source: BMC Bioinformatics. 2011 Oct 14;12:400. doi: 10.1186/1471-2105-12-400 (PMC3213069; doi:10.1186/1471-2105-12-400)
Supplement: Additional file 2 — Supplemental information. The file contains additional pictures representing ROC curves and accuracy variability depending on result list cut-offs for experimental data sets EXP1 and EXP2 and for synthetic data sets SYN5_s, SYN5_f and SYN5_p. [file 1471-2105-12-400-S2.PDF]

Supplemental information – tables

Table 1S. Results of assignment of spectra obtained for selected metabolites from HMDB (data sets SYN1 - SYN4). The assignment was performed with MetaboHunter and HMDB. Search was performed starting from the complete spectra as well as the peak lists. In the examples we have selected metabolites with highly involved spectra and with overlapping peaks with other molecules in the database. Presented are the search scores, number of peaks found relative to the number of peaks in the database (in brackets) and finally the rank of the metabolite in the complete list of found metabolites (bold). Results from searches with MetaboHunter (MH1: Highest number of matched peaks method; MH3: Greedy selection of metabolites method) and HMDB are shown. Noise threshold was set to 1. Note: **NF** = not found; **NR** = not reported.

**Metabolite ID**	**Metabolite name**	**Spectral Search (_s)**	**Spectral Search (_s)**	**Spectral Search (_s)**	**Spectral Search (_s)**	**Peaks search (_f)**	**Peaks search (_f)**	**Peaks search (_f)**	**Peaks search (_f)**	**HMDB NMR Search**<br>**%(matched peaks/total peaks)**<br>**Rank**	**Chenomx Profiler**<br>**%(matched peaks/total peaks)**<br>**Rank**
		**MH1_HMDB**<br>**Score(matched peaks/total peaks)**<br>**Rank**	**MH1_MMCD**<br>**Score(matched peaks/total peaks)**<br>**Rank**	**MH3_HMDB**<br>**Score(matched peaks/total peaks)**<br>**Rank**	**MH3_MMCD**<br>**Score(matched peaks/total peaks)**<br>**Rank**	**MH1_HMDB**<br>**Score(matched peaks/total peaks)**<br>**Rank**	**MH1_MMCD**<br>**Score(matched peaks/total peaks)**<br>**Rank**	**MH3_HMDB**<br>**Score(matched peaks/total peaks)**<br>**Rank**	**MH3_MMCD**<br>**Score(matched peaks/total peaks)**<br>**Rank**		
HMDB00895 / MMCD00168	Acetylcholine	67(4/5)<br>**#1**	44(4/8)<br>**#3**	67(4/5)<br>**#1**	44(4/8)<br>**#3**	67(4/5)<br>**#1**	33(3/8)<br>**#4**	67(4/5)<br>**#1**	33(3/8)<br>**#3**	100(5/5)<br>**#3**	NR<br>#3
HMDB00429 / X	17a-Estradiol	94(84/88)<br>**#1**	X	94(84/88)<br>**#1**	X	63(56 / 88)<br>**#12**	X	**NF**	X	**NF**	**NF**
HMDB0067 / X	Cholesterol	96(89/92)<br>**#1**	X	96(89/92)<br>**#1**	X	94(87/92)<br>**#1**	X	94(87/92)<br>**#1**	X	**NF**	**NF**
HMDB00122 / MMCD00013	D-Glucose	97(38/38)<br>**#1**	90(27/29)<br>**#1**	97(38/38)<br>**#1**	90(27/29)<br>**#1**	97(38/38)<br>**#1**	77(23/29)<br>**#1**	97(38/38)<br>**#1**	77(23/29)<br>**#1**	98(48/48)<br>**#16**	NR<br>#1
**ALL CORRECT IDENTIFICATIONS / TOTAL**		**4 / 4**	**2 / 2**	**4 / 4**	**2 / 2**	**4 / 4**	**2 / 2**	**3 / 4**	**2 / 2**	**2 / 4**	**2/4**

Table 2S. Results of a metabolite search for a spectrum obtained as a combination of spectra for 13 metabolites obtained from HMDB (SYN5). Spectra of individual metabolites were corrected to equal concentration (115mM). Results from searches with MetaboHunter (MH1: Highest number of matched peaks method; MH3: Greedy selection of metabolites method) and HMDB are outlined. Noise threshold was set to 300.

Metabolite ID	Metabolite	Spectral search (_s)	Spectral search (_s)	Spectral search (_s)	Spectral search (_s)	Peaks search (_f)	Peaks search (_f)	Peaks search (_f)	Peaks search (_f)	HMDB NMR Search<br><br>%(matched peaks/total peaks)<br><br>Rank
		MH1_HMDB<br><br>Score(matched peaks/total peaks)<br><br>Rank	MH1_MMCD<br><br>Score(matched peaks/total peaks)<br><br>Rank	MH3_HMDB<br><br>Score(matched peaks/total peaks)<br><br>Rank	MH3_MMCD<br><br>Score(matched peaks/total peaks)<br><br>Rank	MH1_HMDB<br><br>Score(matched peaks/total peaks)<br><br>Rank	MH1_MMCD<br><br>Score(matched peaks/total peaks)<br><br>Rank	MH3_HMDB<br><br>Score(matched peaks/total peaks)<br><br>Rank	MH3_MMCD<br><br>Score(matched peaks/total peaks)<br><br>Rank	
HMDB00097 / MMCD00287	Choline	88(7/7)<br>**#2**	75(6/7)<br>**#16**	62(5/7)<br>**#8**	50(4/7)<br>**#13**	88(7/7)<br>**#5**	75(6/7)<br>**#18**	62(5/7)<br>**#9**	50(4/7)<br>**#13**	**NF**
HMDB00125 / MMCD00162	Glutathione	76(22/28)<br>**#13**	79(19/23)<br>**#11**	**NF**	**NF**	76(22/28)<br>**#20**	83(20/23)<br>**#6**	**NF**	**NF**	**NF**
HMDB00161 / MMCD00067	L-Alanine	71(5/6)<br>**#30**	57(4/6)<br>**#80**	**NF**	**NF**	86(6/6)<br>**#6**	71(5/6)<br>**#28**	29(2/6)<br>**#22**	**NF**	**NF**
HMDB00148 / MMCD00076	L-Glutamic Acid	82(18/21)<br>**#8**	75(12/15)<br>**#15**	**NF**	69(11/15)<br>**#9**	91(20/21)<br>**#1**	88(14/15)<br>**#3**	91(20/21)<br>**#1**	56(9/15)<br>**#10**	**NF**
HMDB00641 / MMCD00077	L-Glutamine	86(18/20)<br>**#3**	77(10/12)<br>**#13**	86(18/20)<br>**#2**	**NF**	90(19/20)<br>**#2**	92(12/12)<br>**#1**	**NF**	92(12/12)<br>**#1**	**NF**
HMDB00687 / MMCD00081	L-Leucine	88(15/16)<br>**#1**	82(9/10)<br>**#4**	88(15/16)<br>**#1**	73(8/10)<br>**#5**	88(15/16)<br>**#4**	82(9/10)<br>**#7**	82(14/16)<br>**#5**	73(8/10)<br>**#6**	**NF**
HMDB00883 / MMCD00091	L-Valine	85(11/12)<br>**#6**	82(9/10)<br>**#3**	85(11/12)<br>**#4**	55(6/10)<br>**#11**	85(11/12)<br>**#8**	73(8/10)<br>**#22**	85(11/12)<br>**#3**	27(3/10)<br>**#25**	**NF**
HMDB00168 / MMCD00069	L-Asparagine	85(11/12)<br>**#7**	77(10/12)<br>**#14**	85(11/12)<br>**#5**	46(6/12)<br>**#17**	85(11/12)<br>**#9**	77(10/12)<br>**#15**	85(11/12)<br>**#4**	46(6/12)<br>**#16**	**NF**

Metabolite ID	Metabolite	Spectral search (_s)	Spectral search (_s)	Spectral search (_s)	Spectral search (_s)	Peaks search (_f)	Peaks search (_f)	Peaks search (_f)	Peaks search (_f)	HMDB NMR Search<br>%(matched peaks/total peaks)<br>Rank
HMDB00172 / MMCD00080	L-Isoleucine	73(22/29)<br>**#21**	63(12/18)<br>**#49**	30(9/29)<br>**#19**	42(8/18)<br>**#19**	73(22/29)<br>**#24**	63(12/18)<br>**#57**	20(6/29)<br>**#28**	42(8/18)<br>**#17**	**NF**
HMDB00190 / MMCD00181	L-Lactic Acid	71(5/6)<br>**#31**	50(7/13)<br>**#126**	43(3/6)<br>**#14**	**NF**	71(5/6)<br>**#37**	50(7/13)<br>**#132**	43(3/6)<br>**#15**	**NF**	**NF**
HMDB00162 / MMCD00086	L-Proline	85(29/33)<br>**#5**	61(17/27)<br>**#59**	71(24/33)<br>**#7**	**NF**	88(30/33)<br>**#3**	68(19/27)<br>**#37**	**NF**	**NF**	**NF**
HMDB00254 / X	Succinic Acid	50(1/1)<br>**#153**	X	**NF**	X	50(1/1)<br>**#156**	X	50(1/1)<br>**#13**	X	**NF**
HMDB00251 / MMCD00122	Taurine	86(6/6)<br>**#4**	71(5/6)<br>**#23**	86(6/6)<br>**#3**	71(5/6)<br>**#6**	86(6/6)<br>**#7**	71(5/6)<br>**#24**	86(6/6)<br>**#2**	71(5/6)<br>**#7**	**NF**
**ALL CORRECT IDENTIFICATIONS / TOTAL**		**13 / 13**	**12 / 12**	**9 / 13**	**7 / 12**	**13 / 13**	**12 / 12**	**10 / 13**	**8 / 12**	**0 / 13**

Table 3S. Experimental mixture of 5 metabolites measured with a 270 MHz spectrometer (EXP2). The list of metabolites and their concentrations as well as the results from searches using MetaboHunter (MH1: Highest number of matched peaks method; MH3: Greedy selection of metabolites method) and HMDB are provided. Noise threshold was set to 10,000.

Metabolite ID	mM	Spectral Search (_s)	Spectral Search (_s)	Spectral Search (_s)	Spectral Search (_s)	Peak Search (_f)	Peak Search (_f)	Peak Search (_f)	Peak Search (_f)	HMDB NMR Search %(matched peaks/total peaks) Rank
		MH1_HMDB Score(matched peaks/total peaks) Rank	MH1_MMCD Score(matched peaks/total peaks) Rank	MH3_HMDB Score(matched peaks/total peaks) Rank	MH3_MMCD Score(matched peaks/total peaks) Rank	MH1_HMDB Score(matched peaks/total peaks) Rank	MH1_MMCD Score(matched peaks/total peaks) Rank	MH3_HMDB Score(matched peaks/total peaks) Rank	MH3_MMCD Score(matched peaks/total peaks) Rank	
Creatine	**4.04179**	67(2/2) **#41**	67(2/2) **#28**	67(2/2) **#2**	67(2/2) **#3**	33(1/2) **#7**	33(1/2) **#4**	**NF**	33(1/2) **#3**	2/2 **#3**
D-Glucose	**5.2397**	95(37/38) **#1**	93(28/29) **#1**	95(37/38) **#1**	93(28/29) **#1**	26(10/38) **#15**	17(5/29) **#38**	**NF**	**NF**	27/48 **#33**
Citric acid	**1.06704**	40(2/4) **#154**	X	40(2/4) **#14**	X	20(1/4) **#46**	X	20(1/4) **#7**	X	3/4 **#16**
Phosphocholine	**0.20411**	33(3/8) **#215**	X	**NF**	X	**NF**	X	**NF**	X	**NF**
Acetylcholine	**0.10789**	67(4/5) **#40**	*57(4/6) **#52**	**NF**	**NF**	17(1/5) **#69**	**NF**	**NF**	**NF**	5/5 **#10**
**ALL CORRECT IDENTIFICATIONS / TOTAL**		**5 / 5**	**3 / 3**	**3 / 5**	**2 / 3**	**3 / 5**	**2 / 3**	**1 / 5**	**1 / 3**	**4 / 5**

* MMCD includes the *Acetylcholine perchlorate* metabolite (expnmr_00381) which is the closest match to the *Acetylcholine* metabolite (HMDB00895) from HMDB.

Table 4S. Results of metabolite search for a combination of HMDB-extracted peak lists corresponding to a mixture of 13 metabolites (SYN5).

Metabolite ID	Metabolite name	Peaks search using HMDB peaks info (_p)	Peaks search using HMDB peaks info (_p)	Peaks search using HMDB peaks info (_p)	Peaks search using HMDB peaks info (_p)	HMDB NMR search using peak list data<br>%(matched peaks/total peaks)<br>Rank
		**MH1_HMDB**<br>Score(matched peaks/total peaks)<br>Rank	**MH1_MMCD**<br>Score(matched peaks/total peaks)<br>Rank	**MH3_HMDB**<br>Score(matched peaks/total peaks)<br>Rank	**MH3_MMCD**<br>Score(matched peaks/total peaks)<br>Rank	
HMDB00097 / MMCD00287	Choline	88(7/7)<br>**#9**	75(6/7)<br>**#10**	88(7/7)<br>**#5**	75(6/7)<br>**#3**	100(13/13)<br>**#36**
HMDB00125 / MMCD00162	Glutathione	97(28/28)<br>**#3**	83(20/23)<br>**#6**	97(28/28)<br>**#2**	71(17/23)<br>**#7**	100(31/31)<br>**#32**
HMDB00161 / MMCD00067	L-Alanine	86(6/6)<br>**#11**	86(6/6)<br>**#5**	**NF**	**NF**	100(6/6)<br>**#10**
HMDB00148 / MMCD00076	L-Glutamic Acid	95(21/21)<br>**#4**	94(15/15)<br>**#1**	**NF**	94(15/15)<br>**#1**	100(30/30)<br>**#18**
HMDB00641 / MMCD00077	L-Glutamine	95(20/20)<br>**#5**	92(12/12)<br>**#2**	**NF**	**NF**	100(28/28)<br>**#7**
HMDB00687 / MMCD00081	L-Leucine	94(16/16)<br>**#6**	73(8/10)<br>**#12**	94(16/16)<br>**#3**	55(6/10)<br>**#10**	(23/25)<br>**#45**
HMDB00883 / MMCD00091	L-Valine	92(12/12)<br>**#7**	82(9/10)<br>**#7**	92(12/12)<br>**#4**	82(9/10)<br>**#2**	100(16/16)<br>**#8**
HMDB00168 / MMCD00069	L-Asparagine	92(12/12)<br>**#8**	69(9/12)<br>**#19**	85(11/12)<br>**#6**	46(6/12)<br>**#14**	100(12/12)<br>**#31**
HMDB00172 / MMCD00080	L-Isoleucine	97(29/29)<br>**#2**	79(15/18)<br>**#8**	73(22/29)<br>**#7**	74(14/18)<br>**#4**	100(42/42)<br>**#35**
HMDB00190 / MMCD00216	L-Lactic Acid	86(6/6)<br>**#12**	71(5/6)<br>**#16**	43(3/6)<br>**#11**	**NF**	100(6/6)<br>**#12**
HMDB00162 / MMCD00086	L-Proline	97(33/33)<br>**#1**	71(20/27)<br>**#13**	97(33/33)<br>**#1**	**NF**	(39/44)<br>**#49**
HMDB00254 / X	Succinic Acid	50(1/1)	X	**NF**	X	(1/1)

Metabolite ID	Metabolite name	Peaks search using HMDB peaks info (_p)	Peaks search using HMDB peaks info (_p)	Peaks search using HMDB peaks info (_p)	Peaks search using HMDB peaks info (_p)	HMDB NMR search using peak list data<br>%(matched peaks/total peaks)<br>Rank
		#79				#11
HMDB00251 / MMCD00122	Taurine	86(6/6)<br>#13	71(5/6)<br>#14	29(2/6)<br>#12	71(5/6)<br>#5	NF
ALL CORRECT IDENTIFICATIONS / TOTAL		13 / 13	12 / 12	9 / 13	8 / 12	12 / 13

Table 5S. Results of metabolite search for the ^{1}H-NMR spectrum of a spike-in urine sample provided by (Zheng et al., 2011) and measured on a 500 MHz Brucker Avance NMR spectrometer (EXP1). The symbol "X" means the metabolite does not exist in the database. Noise threshold was set to 5.

Metabolite ID	Metabolite name	Peaks search using HMDB peaks info (_p)				HMDB NMR search using peak list data<br>%(matched peaks/total peaks)<br>Rank
		MH1_HMDB<br>Score(matched peaks/total peaks)<br>Rank	MH1MMCD<br>Score(matched peaks/total peaks)<br>Rank	MH3_HMDB<br>Score(matched peaks/total peaks)<br>Rank	MH3_MMCD<br>Score(matched peaks/total peaks)<br>Rank	
**Spiked-in metabolites**	**Spiked-in metabolites**	**Spiked-in metabolites**	**Spiked-in metabolites**	**Spiked-in metabolites**	**Spiked-in metabolites**	**Spiked-in metabolites**
HMDB00251 / MMCD00122	Taurine	57(4/6)<br>**#84**	57(4/6)<br>**#64**	**NF**	43(3/6)<br>**#16**	**NF**
HMDB00714 / MMCD00378	Hippuric acid	54(7/12)<br>**#96**	30(3/9)<br>**#208**	46(6/12)<br>**#21**	**NF**	**NF**
HMDB06809 / MMCD00101	Nicotinate	**NF**	23(7/29)<br>**#241**	**NF**	**NF**	**NF**
HMDB00744 / MMCD00083	Malic acid	15(2/12)<br>**#590**	17(2/11)<br>**#281**	**NF**	9(1/10)<br>**#39**	**NF**
HMDB00208 / MMCD00021	Oxoglutaric acid	29(2/6)<br>**#330**	57(4/6)<br>**#67**	56(5/8)<br>**#12**	57(4/6)<br>**#9**	71(5/6)<br>**#91**
**Other metabolites commonly found in urine (Gronwald et al., 2008)**	**Other metabolites commonly found in urine (Gronwald et al., 2008)**	**Other metabolites commonly found in urine (Gronwald et al., 2008)**	**Other metabolites commonly found in urine (Gronwald et al., 2008)**	**Other metabolites commonly found in urine (Gronwald et al., 2008)**	**Other metabolites commonly found in urine (Gronwald et al., 2008)**	**Other metabolites commonly found in urine (Gronwald et al., 2008)**
HMDB00042 / X	Acetic acid	**NF**	X	**NF**	X	**NF**
HMDB00161 / MMCD00067	L-Alanine	43(3/6)<br>**#174**	57(4/6)<br>**#55**	**NF**	**NF**	71(5/6)<br>**#88**
X / MMCD00402	3-aminoisobutyrate	X	43(6/13)<br>**#134**	X	**NF**	X
HMDB00670 / MMCD00068	L-Arginine	28(11/38)<br>**#336**	29(5/16)<br>**#210**	**NF**	**NF**	**NF**

Metabolite ID	Metabolite name	Peaks search using HMDB peaks info (_p)				HMDB NMR search using peak list data<br>%(matched peaks/total peaks)<br>Rank
X / MMCD00028	Betaine	X	67(2/2)<br>**#30**	X	67(2/2)<br>**#7**	100(2/2)<br>**#19**
HMDB00562 / MMCD00156	Creatinine	33(1/2)<br>**#279**	75(3/3)<br>**#15**	**NF**	**NF**	100(2/2)<br>**#24**
HMDB00149 / MMCD00278	Ethanolamine	43(3/6)<br>**#173**	43(3/6)<br>**#135**	43(3/6)<br>**#22**	**NF**	100(7/7)<br>**#41**
HMDB00641 / MMCD00077	L-Glutamine	52(11/20)<br>**#105**	54(7/12)<br>**#80**	**NF**	**NF**	(23/28)<br>**#94**
HMDB00123 / MMCD00057	Glycine	50(1/1)<br>**#135**	50(1/1)<br>**#109**	**NF**	50(1/1)<br>**#13**	100(1/1)<br>**#18**
HMDB00177 / MMCD00078	L-Histidine	50(7/13)<br>**#113**	60(9/14)<br>**#49**	**NF**	**NF**	**NF**
HMDB00182 / MMCD00082	L-Lysine	26(8/30)<br>**#377**	22(4/17)<br>**#248**	**NF**	**NF**	**NF**
HMDB00001 / MMCD00318	1-methylhistidine	40(6/14)<br>**#199**	50(8/15)<br>**#99**	**NF**	**NF**	**NF**
HMDB00479 / X	3-methylhistidine	60(9/14)<br>**#73**	X	**NF**	**NF**	(13/15)<br>**#77**
HMDB00925 / MMCD00323	Trimethyl-N-oxide	50(1/1)<br>**#132**	50(1/1)<br>**#108**	**NF**	**NF**	100(1/1)<br>**#35**
**ALL CORRECT IDENTIFICATIONS / TOTAL**		**15 / 17**	**17 / 17**	**3 / 17**	**5 / 17**	**8 / 17**

Table 6S. Accuracies for cut-off = mixture size for SYN5, EXP1 and EXP2 data sets.

Data set	MH1_HMDB	MH1_MMCD	MH3_HMDB	MH3_MMCD	HMDB NMR Search
**SYN5_s**	**0.991**	0.960	0.986	0.964	0.974
**SYN5_f**	**0.991**	0.960	0.986	0.964	0.972
**SYN5_p**	**0.998**	0.982	0.991	0.973	0.983
**EXP1**	0.970	0.942	0.970	0.946	**0.972**
**EXP2**	0.972	0.946	0.975	0.951	**0.976**
